# Genome-wide and protein kinase-focused RNAi screens reveal conserved and novel damage response pathways in *Trypanosoma brucei*

**DOI:** 10.1371/journal.ppat.1006477

**Published:** 2017-07-24

**Authors:** Jennifer A. Stortz, Tiago D. Serafim, Sam Alsford, Jonathan Wilkes, Fernando Fernandez-Cortes, Graham Hamilton, Emma Briggs, Leandro Lemgruber, David Horn, Jeremy C. Mottram, Richard McCulloch

**Affiliations:** 1 The Wellcome Centre for Molecular Parasitology, Institute of Infection, Immunity and Inflammation, University of Glasgow, Glasgow, United Kingdom; 2 The London School of Hygiene and Tropical Medicine, London, United Kingdom; 3 Glasgow Polyomics, Wolfson Wohl Cancer Research Centre, University of Glasgow, Garscube Estate, Bearsden, United Kingdom; 4 Division of Biological Chemistry & Drug Discovery, School of Life Sciences, University of Dundee, Dundee, United Kingdom; 5 Centre for Immunology and Infection, Department of Biology, University of York, York, United Kingdom; Marine Biological Laboratory, UNITED STATES

## Abstract

All cells are subject to structural damage that must be addressed for continued growth. A wide range of damage affects the genome, meaning multiple pathways have evolved to repair or bypass the resulting DNA lesions. Though many repair pathways are conserved, their presence or function can reflect the life style of individual organisms. To identify genome maintenance pathways in a divergent eukaryote and important parasite, *Trypanosoma brucei*, we performed RNAi screens to identify genes important for survival following exposure to the alkylating agent methyl methanesulphonate. Amongst a cohort of broadly conserved and, therefore, early evolved repair pathways, we reveal multiple activities not so far examined functionally in *T*. *brucei*, including DNA polymerases, DNA helicases and chromatin factors. In addition, the screens reveal *Trypanosoma*- or kinetoplastid-specific repair-associated activities. We also provide focused analyses of repair-associated protein kinases and show that loss of at least nine, and potentially as many as 30 protein kinases, including a nuclear aurora kinase, sensitises *T*. *brucei* to alkylation damage. Our results demonstrate the potential for synthetic lethal genome-wide screening of gene function in *T*. *brucei* and provide an evolutionary perspective on the repair pathways that underpin effective responses to damage, with particular relevance for related kinetoplastid pathogens. By revealing that a large number of diverse *T*. *brucei* protein kinases act in the response to damage, we expand the range of eukaryotic signalling factors implicated in genome maintenance activities.

## Author summary

Damage to the genome is a universal threat to life. Though the repair pathways used to tackle damage can be widely conserved, lineage-specific specialisations are found, reflecting the differing life styles of extant organisms. Using RNAi coupled with next generation sequencing we have screened for genes that are important for growth of *Trypanosoma brucei*, a diverged eukaryotic microbe and important parasite, in the presence of alkylation damage caused by methyl methanesulphonate. We reveal both repair pathway conservation relative to characterised eukaryotes and specialisation, including uncharacterised roles for translesion DNA polymerases, DNA helicases and chromatin factors. Furthermore, we demonstrate that loss of around 15% of *T*. *brucei* protein kinases sensitises the parasites to alkylation, indicating phosphorylation signalling plays widespread and under-investigated roles in the damage response pathways of eukaryotes.

## Introduction

Faithful genome transmission is necessary for the growth and propagation of all organisms. Damage to the genome can arise from a myriad of sources, including exposure to mutagenic chemicals and metabolic or replicative by-products. If damage is left unrepaired, the genetic information can be altered, leading to death, reduced fecundity and disease in multicellular organisms. To counter all potential genotoxic lesions, a wide range of DNA repair pathways, collectively known as the DNA damage response (DDR), are found in all three domains of life, though with variation in the underlying machineries of each pathway and their relative use in different organisms [1, 2]. More widely, genome repair is one arm of a range of processes that allow cells to limit or tackle cellular damage.

*Trypanosoma brucei* is an extracellular protozoan parasite of mammals, causing the neglected disease African trypanosomiasis (sleeping sickness in humans, Nagana in cattle)[3]. In common with related kinetoplastids, *T*. *brucei* shows divergence in several core cellular processes, including the near universal use of multigenic transcription and reliance on post-transcriptional strategies for gene expression control. Nonetheless, *T*. *brucei* is a genetically tractable protozoan, making it a valuable model amongst eukaryotic microbes. Multiple DDR pathways operate in kinetoplastids, including three forms of excision repair (mismatch, nucleotide and base) and at least two forms of DNA break repair (homology- and microhomology-directed)[1, 4]. Non-homologous end-joining (NHEJ), an important break repair pathway in all domains of life, appears to be absent in kinetoplastids, despite the presence of both subunits of the Ku heterodimer [5–9]. Furthermore, homologous recombination (HR) not only provides for DNA break repair genome-wide, but also catalyses the locus-directed movement of Variant Surface Glycoprotein (VSG) genes that underpins immune evasion by antigenic variation in *T*. *brucei* [10]. The above knowledge has been accrued through homology-informed candidate gene studies, meaning several DDR activities have not been functionally tested and potentially kinetoplastid-specific activities may have escaped detection. Virtually no work has examined how the DDR, cell and life cycle progression are linked in kinetoplastids. Protein kinase (PK) signalling is likely to play a central role in such links. However, no work has described any PK that acts in the kinetoplastid DDR, despite phosphorylation of several *T*. *brucei* repair proteins, including BRCA2 and RAD50, having been described [11], though the functional significance of the modifications is unknown. Damage-dependent phosphorylation of *T*. *brucei* histone H2A on Thr130, generating the kinetoplastid variant of the γH2A(X) chromatin modification [12], has also been described [13], but the parasite PK(s) that directs this alteration and its contribution to repair has not been examined. These gaps in understanding of PK signalling and wider aspects of the kinetoplastid DDR are impediments to understanding the evolution of the eukaryotic DDR and to evaluating the potential anti-parasite efficacy of compounds that target repair-associated factors, such as anti-cancer approaches acting on the phosphatidyl inositol 3-kinase-like PKs ATR and ATM [14, 15], which play key roles in recognising DNA breaks and directing the appropriate repair pathway, and have homologues in *T*. *brucei*.

To identify the full complement of gene products and pathways that act in damage repair, comprehensive screens are needed, such as have been deployed in characterising the DDR in other eukaryotes [16]. In *T*. *cruzi*, changes in RNA [17] and protein [18] levels after exposure to ionizing radiation have been assessed, but genome-wide screening of kinetoplastid mutants after exposure to damage has not been attempted. RNAi coupled with next generation sequencing, termed RNAi target sequencing (RIT-seq), has been shown to be a feasible approach to evaluate the importance of potentially all genes in *T*. *brucei* during growth and differentiation [19]. Subsequent RIT-seq screens have identified genes involved in anti-trypanosome drug action [20–22], human serum susceptibility [23] and quorum-sensing [24], in each case by selecting for cells in the population that can grow in the presence of selection only after RNAi. To date, RIT-seq has not been used to screen for *T*. *brucei* genes whose loss by RNAi increases sensitivity to a treatment. Here, we describe such a ‘synthetic lethal’ RIT-seq approach, seeking to identify genes whose loss sensitises *T*. *brucei* to methyl methanesulphonate (MMS), an Sn2 alkylator [25]. MMS causes DNA lesions, including breaks, which can be toxic, mutagenic and prevent DNA synthesis by impeding replication fork movement. The transcriptional and proteomic responses of several eukaryotic cells to MMS have been described, revealing wide-ranging changes suggestive of a network of adaptations to cope with MMS-induced damage, some common to other types of DNA damage and stress [26]. In addition, three studies, two in yeast using gene mutants [27, 28] and one in *Drosophila melanogaster* using RNAi [29], have described genes involved in MMS tolerance and confirm that multiple pathways, including DDR reactions, contribute to the response to this widely used genotoxic agent.

RIT-seq screening of MMS-treated bloodstream form (BSF) *T*. *brucei* described here revealed several MMS damage response pathways, including homologous recombination and nucleotide excision repair, which are common between the kinetoplastid parasite, yeast and *D*. *melanogaster*, though at least two pathways appear not to act in *T*. *brucei*: transcriptional control and Notch signalling Several of the conserved MMS damage response pathways we reveal have not been examined previously. In addition, many putative *T*. *brucei*-specific MMS repair-associated proteins are revealed whose functions could not have been evaluated previously, as they lack sequence homology with other eukaryotes. Finally, a focus on PKs revealed 30 proteins (many of which appear essential) whose loss is predicted to sensitise BSF *T*. *brucei* cells to MMS. We provide targeted validation of nine novel *T*. *brucei* PKs that act in MMS damage response, including detailed analysis of an aurora PK. The range of PK families uncovered exceeds the PKs previously implicated in the eukaryotic damage response, suggesting unanticipated functions. The two screens therefore provide insight into cellular repair activities in *T*. *brucei*, some novel and some likely conserved in other eukaryotes.

## Results and discussion

### A genome-wide RNAi screen for *T*. *brucei* MMS damage response factors

**Fig 1:**
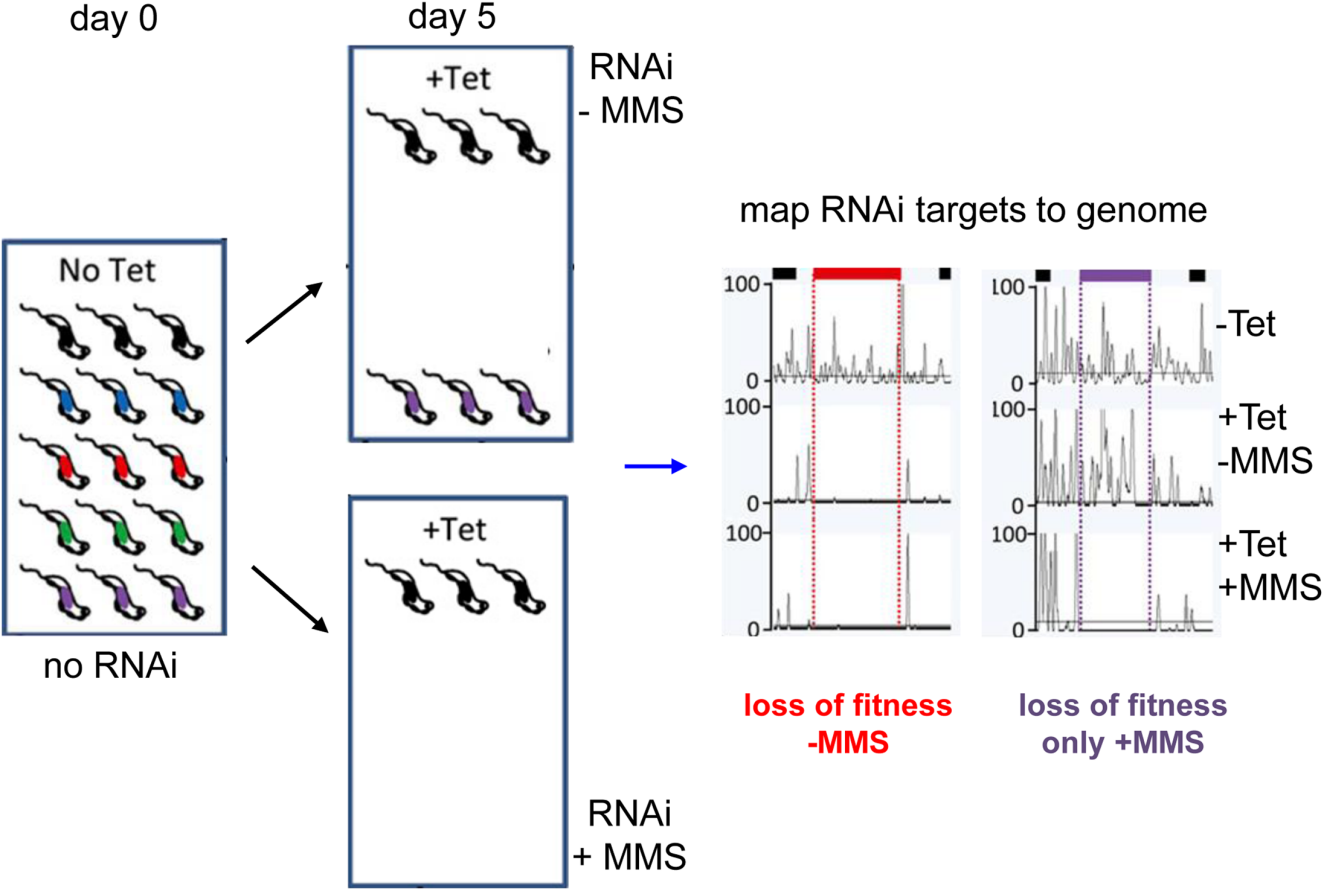
Schematic outline of the whole genome *T*. *brucei* MMS RIT-seq screen. A whole genome tetracycline (Tet) inducible RNAi library was established in BSF *T*. *brucei* cells as a pool, within which random RNAi fragments target potentially all genes and provide unique identifiers. Cells were induced by Tet addition (+) for a total of 5 days, during which cells targeting RNAi against important genes (red, green, blue) are lost from or reduced in the population. In parallel, Tet+ cells were grown in the presence of methyl methanesulphonate (MMS, 0.0003%), which was added 1 day after RNAi induction. Cells carrying an RNAi target for a gene necessary for repair of MMS damage (purple) are specifically lost or depleted in the Tet+, MMS+ population relative to the Tet+, MMS- population. PCR was used to amplify all RNAi target fragments after five days of RNAi with or without exposure to MMS; the amplicons were sequenced and mapped to the genome. Read depth mapping is shown schematically for a gene whose RNAi causes loss of fitness without MMS (red), and for a gene whose RNAi causes loss of fitness only after MMS exposure (purple).

We used BSF *T*. *brucei* cells, the life cycle stage that causes mammalian disease, to run a RIT-seq screen for MMS damage response factors (Fig 1). To this end, an RNAi fragment library representing >99% of the genome in a population of ~ 10 million cells [19, 30] was grown for 24 hours (3–4 population doublings) in the presence of tetracycline (Tet), which induces RNAi (Fig 1). Genomic DNA was prepared from a sample of the population, which was then split into four cultures and allowed to grow for another four days in the presence of Tet. Two of the cultures were grown throughout the four days in 0.0003% MMS, a concentration that induces damage (as evidenced by increased γH2A levels)[13] and impairs, but does not prevent, growth (see below). Genomic DNA was then prepared from all four BSF populations (subjected to RNAi for a total of 5 days). By mapping loss of gene-specific reads in cells that were both RNAi induced and MMS-treated relative to cells subjected to RNAi but not to MMS, we sought to identify genes specifically required to maintain growth in the presence of MMS induced damage. To do so, we PCR-amplified the RNAi targets using primers that flank all RNAi constructs integrated into the genome [31] and limited cycle PCR. The PCR resulted in a range of products between ~0.2–1.6 kbp in all samples (S1 Fig) that reflects the sizes of the RNAi target fragments in the RNAi library [30]. The PCR products were then sequenced and reads were mapped to a ‘minimal’ version of the *T*. *brucei* genome that included only the 9849 predicted CDS, with a comparable read depth profile to a previous RITseq after RNAi alone (S2 Fig)[19].

**Fig 2:**
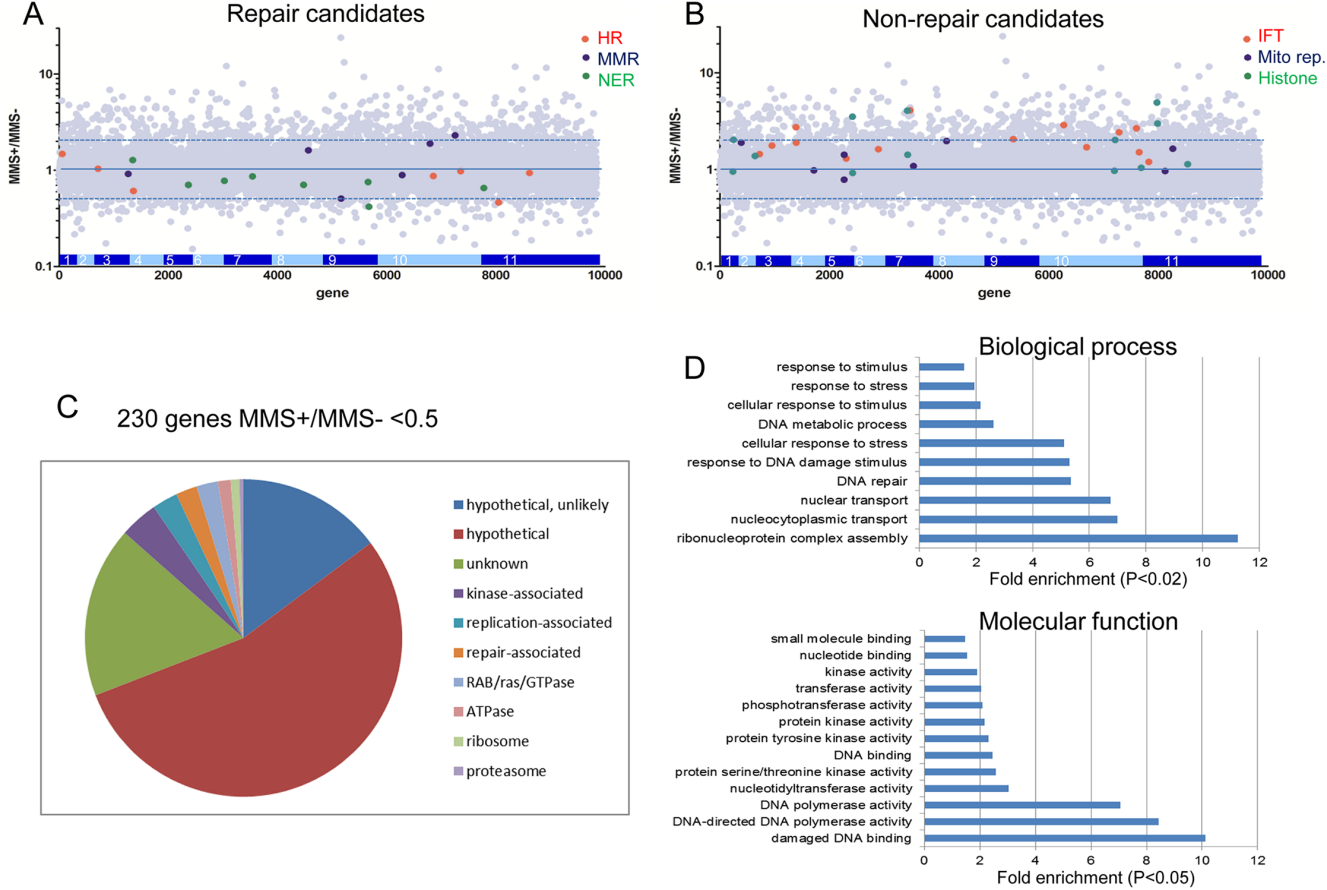
Analysis of the MMS RIT-seq screen. **A, B.** Scatter plots showing the ratio of mapped RNAi target-specific reads for every gene (grey dots) in the RNAi-induced, MMS-treated population relative to the RNAi-induced, untreated population (MMS+/MMS-); gene location within the 11 megabase chromosomes is shown and dotted lines indicate 2-fold increase and decrease in MMS+/MMS- ratio. Genes are highlighted with roles in (A) homologous recombination (HR, red), mismatch repair (MMR, blue) and nucleotide excision repair (NER, green), or in (B) intraflagellar transport (IFT, red), mitochondrial replication (Mito rep, blue) and encoding histones (green). **C.** A pie chart of the distribution of all genes displaying an MMS+/MMS- ratio of less than 0.5, excluding 44 genes predicted to be VSGs. Hypothetical and hypothetical unlikely denotes genes for which there are currently no homology-predicted functions. Unknown denotes genes with homology-predicted functions that cannot be readily associated with the response to MMS damage. Finally, genes in seven classes of predicted functions with putative roles in responding to MMS are detailed. **D.** GO terms, within two headings, which show significantly increased frequency in the MMS+/MMS- <0.5 gene set relative to the whole GO gene set (IDs and further analysis are provided in S3 Table).

Fig 2 shows an evaluation of the effect of MMS on gene abundance in the population after 5 days of RNAi-induction. For each sample, the number of sequence reads that mapped to every annotated gene was determined and normalised relative to CDS length and total number of reads in the library. These read depth values were then averaged for the two Tet+, MMS- samples and for the two Tet+, MMS+ samples, and the ratio of reads in the latter determined relative to the former. The resulting MMS+/MMS- ratio for every gene was viewed in a scatter plot relative to gene position on the 11 *T*. *brucei* chromosomes (Fig 2A and 2B; individual gene data in S1 Table). Given the limitations of having only duplicate samples at one control and one experimental time point using a single concentration of MMS, we consider it likely that the screen is most robust when considering read depth trends across damage response pathways or networks, and should be viewed with caution when comparing read depth to evaluate the roles of individual genes. Thus, we first examined cohorts of genes characterised to act in three DNA repair pathways (Fig 2A). HR and nucleotide excision repair (NER) pathways have been extensively characterised in *T*. *brucei* and have been implicated in the MMS damage response in *Drosophila* and yeast [29]. MMS+/MMS- ratios for multiple HR and NER genes whose functions have been examined previously revealed a trend towards <1 (five of seven HR genes, seven of eight NER genes; S2 Table), indicating that cells in which RNAi targets these genes are depleted in the MMS-treated population relative to the untreated control. Indeed, the MMS+/MMS- ratios of the NER genes matched what is known regarding the novel operation of this repair pathway. Specifically, global genome NER in *T*. *brucei* has undergone neofunctionalisation to act in an essential genome repair pathway, manifesting as increased sensitivity to inter-strand crosslink damage upon depletion of these NER factors [32]. NER in response to transcription stalling (transcription-coupled NER) is the main form of NER in *T*. *brucei* and bypasses recruitment of TFIIH-associated XPB and XPD helicases, using instead a novel XPB orthologue (XPBz)[33]. Thus, *T*. *brucei* XPBz registered an MMS+/MMS- ratio of 1.36, consistent with the lack of MMS sensitivity previously described in *xpbz* null mutants [33]. In contrast, XPC (the lesion recognition factor during global genome NER) showed the lowest MMS+/MMS- ratio (~0.41), suggesting a repair function involving recruitment of the XPB and XPD helicases (consistent with MMS sensitivity following RNAi of TFIIH components)[33, 34] and the XPF/ERCC1 endonuclease complex; all of these factors had MMS+/MMS- ratios of <0.75. Ratios of the transcription-coupled NER factors CSB and XPG (0.86 and 0.75) were higher than for global genome NER factors, suggesting this NER pathway plays a lesser role in the response to MMS. Overall, the RIT-seq outputs suggest global NER plays a more profound role in response to MMS genome damage than HR, since the MMS+/MMS- ratios of most HR factors were rarely as low (Fig 2A, S2 Table). The two HR genes with the lowest MMS+/MMS- ratios were RAD51 paralogues, RAD51-3 and RAD51-6 (0.46 and 0.61)[35, 36], perhaps indicating specialised activities, such as those described in *Leishmania* [37] and related to MMS-induced replicative stress [38]. Though mismatch repair (MMR) has been implicated in MMS repair elsewhere [29], the evidence for a similar role in *T*. *brucei* is weak (Fig 2A). Here, three of six annotated MMR genes had MMS+/MMS- ratios <1, none of which have been functionally examined in any kinetoplastid; nonetheless, MSH3 (0.50) is known to contribute to processing of HR strand exchange intermediates in other eukaryotes [39].

**Fig 3:**
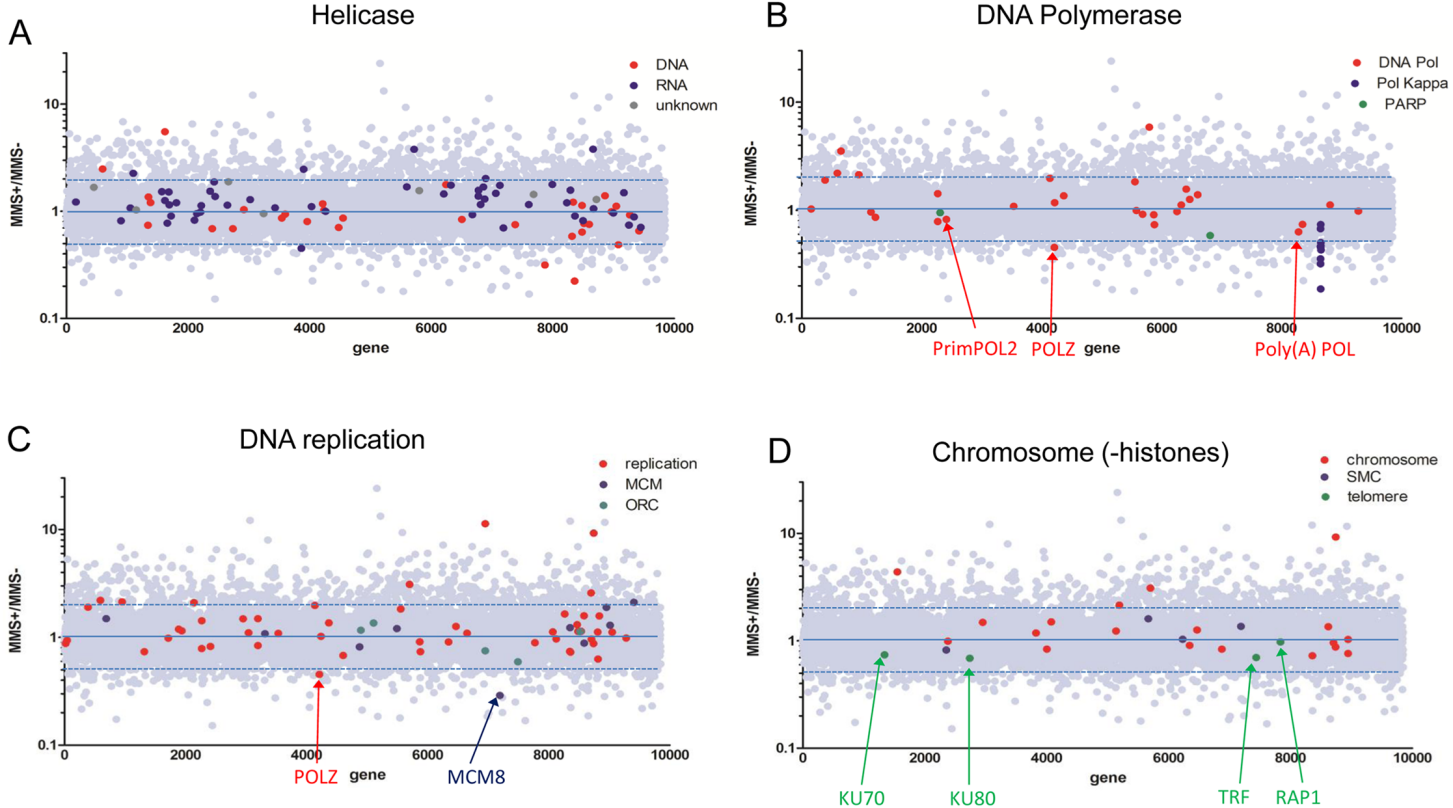
MMS RIT-seq prediction of gene categories providing damage response functions. Scatter plots are shown of MMS+/MMS- read depth ratios for all *T*. *brucei* genes (grey dots), as in Fig 2, highlighting individual genes within four functional categories (further details provided in S2 Table). **A.** All predicted *T*. *brucei* helicase genes are separated into putative RNA (blue) and DNA helicases (red), or those whose substrate is unclear (dark grey). **B.** All genes encoding predicted DNA polymerase (Pol) activities are shown in red, with specific factors arrowed; putative DNA Pol kappa genes are highlighted blue and two Poly (ADP-ribose) Pol genes (PARP) in green. **C.** All genes with predicted involvement in DNA replication are in red; blue highlights Minichromosome Maintenance Complex (MCM)-related factors, and green denotes Origin Recognition Complex (ORC) factors. **D.** Genes with chromosome structure-associated functions are in red, with telomere factors in green and cohesin/condensin (SMC) factors in blue.

Base excision repair (BER) is a key response pathway to MMS damage [29], but evaluating this in *T*. *brucei* is complicated by lack of genetic analyses of most constituent genes and the unusual targeting of two DNA polymerase (Pol) beta homologues to the kinetoplast [40], questioning if and how BER operates in the nucleus. Nevertheless, a role for *T*. *brucei* BER in tackling MMS damage is consistent with six of ten putative BER factors displaying MMS+/MMS- ratios < 1 (S2 Table). Amongst these factors, one DNA glycosylase (OGG1), so far only examined functionally in *T*. *cruzi* [41], showed the strongest evidence for MMS repair, consistent with MMS generating oxidative DNA damage [29]. In addition, one of the two mitochondrial DNA Pol beta genes displayed an MMS+/MMS- ratio of 0.79, though whether this indicates a role in repair of nuclear or kinetoplast damage is unknown [42]. BER also contributes to repair of single strand DNA breaks, which MMS generates. Poly (ADP-ribose) polymerase (PARP) recognises such breaks [43] and, intriguingly, a potentially novel and uncharacterised PARP showed clearer evidence of a repair role (S2 Table; see Fig 3, below) than the more conventional kinetoplastid PARP homologue [44].

Another key factor in responding to genome damage is the heterotrimeric Rad9-Rad1-Hus1 (9-1-1) complex and the MMS+/MMS- ratios of the component genes suggest the *T*. *brucei* complex acts in genome surveillance (S2 Table). Indeed, the subtly different MMS+/MMS- ratios for Rad9 (0.82) and Hus1 (1.08) appear consistent with the different phenotypes of the two mutants in *Leishmania* after exposure to MMS, suggesting the *T*. *brucei* factors may also play distinct roles outside the 9-1-1 complex [45].

The above analysis relies upon a trend for MMS+/MMS- ratios <1 amongst a cohort of DNA repair genes. To test this predictive approach, we examined the MMS+/MMS- ratios in three gene cohorts not expected to act in the MMS damage response (Fig 2B). First, none of 14 genes implicated in intraflagellar transport had an MMS+/MMS- ratio < 1. Second, we examined core and variant histones [46], plotting the maximum and minimum MMS+/MMS- ratios amongst the multigene arrays encoding histones H2A, H2B, H3 and H4; only three of 13 values were < 1, and the lowest was 0.92. Finally, we examined eight proteins implicated in kinetoplast replication (six DNA Pols and two primases); seven of the MMS+/MMS- ratios were 0.97 or above, and the gene with the lowest ratio (0.79) encodes DNA Pol Beta (discussed above).

### Conserved and non-conserved *T*. *brucei* MMS damage response genes and pathways

To examine more broadly how *T*. *brucei* responds to MMS exposure, we selected the 274 genes that had an average MMS+/MMS- ratio of 0.5 or less, indicating 2-fold or greater loss of reads after RNAi in the presence of MMS than the absence. 44 were predicted to encode VSGs or VSG pseudogenes and were discounted as mapping artefacts (S3 Table). Though for the majority of the remaining 230 genes no predicted function is currently available (as they are annotated as hypothetical or hypothetical-unlikely; Fig 2C), we examined what processes are represented in the gene set by asking which gene ontology (GO) terms, in two classifications, displayed significant enrichment (Fig 2D; all significantly enriched GO terms are shown in S3 Table). Enrichment of genes involved in DNA functions was widespread, and the pronounced enrichment of the GO terms ‘DNA repair’ and ‘damaged DNA binding’ (both P values <0.0001) is consistent with the above analysis of known DNA repair pathways (Fig 2, S2 Table). Thus, the cohort of hypothetical genes in this set is likely a rich source of previously undiscovered damage-repair factors.

An RNAi screen in *D*. *melanogaster* predicted 13 pathways that act to tackle MMS damage (S3 Table), with many conserved in yeast and mammals [29]. Six of these pathways involve DNA repair, including the four pathways discussed above. One other pathway was DNA damage signalling initiated by DNA interacting PKs (see below for discussion of kinome-focused RIT-seq). The sixth repair pathway involved RecQ-like helicases, of which *D*. *melanogaster* encodes four. Two RecQ helicases are found in *T*. *brucei*, one of which displayed an MMS+/MMS- ratio of 0.86 (S2 Table), consistent with MMS sensitivity of null mutants [47]. Given the ubiquitous roles of helicases in DNA and RNA biology and the availability of classification and functional predictions for the estimated 112 helicases in *T*. *brucei* [48], we examined the MMS+/MMS- ratios of all putative helicase genes (Fig 3A, S2 Table). Separating the helicases into those most likely to act on DNA or RNA suggested that tackling DNA damage by MMS is more critical, since a larger percentage of DNA helicases (62%) than RNA helicases (32%) displayed MMS+/MMS- ratios <1. Of the 21 DNA helicases with a ratio <1, 11 have yet to be functionally examined (including three with ratios <0.5; Fig 3A, S3 Fig, S2 Table), suggesting the existence of unexplored pathways of genome maintenance. Amongst the characterised DNA helicases are eight Pif1-like helicases, most of which are mitochondrial; five of these genes displayed MMS+/MMS- ratios <1 but separating repair functions from predicted kinetoplast replication roles (PIF1, PIF8) would require further analysis [49–51]. Two genes encoding RuvB-like factors, potentially *T*. *brucei* homologues of Pontin and Reptin in other eukaryotes [52], are notable for displaying >5 fold increased reads in the MMS-treated cells relative to the controls (S1 and S2 Tables). However, analysing genes or gene sets that show enrichment in the presence of MMS is more problematic to interpret in terms of the damage response than enhanced sensitivity, and so we did not explore this category of genes further.

Seven MMS damage response pathways potentially unlinked to DNA repair have been predicted (S3 Table): transcription and translation, ATP and glutathione metabolism, Notch and TOR signalling, and proteasome function [29]. None of these pathways were significantly over-represented in the cohort of 230 genes described above, but only two can be ruled out as damage response strategies in *T*. *brucei* (S3 Table). The Notch pathway acts to determine cell fate [53], but no evidence of this pathway has been described in any kinetoplastid to date. MMS sensitivity as a result of transcription factor loss, as well as changes in the expression of core transcription factor genes after exposure to MMS, has been described in a number of eukaryotes [28, 29, 54]. Thus, the absence of enrichment in GO terms associated with transcription in the MMS+/MMS- <0.5 gene set, despite >200 genes annotated as transcription factors in *T*. *brucei*, appears meaningful. Perhaps the ubiquitous use of multigenic transcription, and the resulting devolution of gene expression controls to post-transcriptional mechanisms in kinetoplastids [55], means *T*. *brucei* cannot respond to MMS stress by upregulating gene transcription. The RNAi approach we have taken to identify MMS damage response factors is likely to under-represent essential genes, since mapped reads would be low after five days of RNAi even in the absence of MMS. Since many components involved in translation, proteasome function and ATP metabolism are essential [19, 56], it is intriguing that a small number of genes (two, one and three, respectively; Fig 2C) involved in each of these functions was detected amongst the 230 genes in the MMS+/MMS- <0.5 set. In this regard, it is notable that transcriptome [17] and proteome [18] analyses indicate that active (and perhaps enhanced) translation is needed to allow *T*. *cruzi* to recover from ionising radiation exposure. In kinetoplastids, many aspects of glutathione metabolism have been usurped by trypanothione, which has a key role in defence against oxidative damage [57], and MMS+/MMS- ratios of component genes (S2 Table; three of eight genes <1) may suggest this novel pathway contributes to the *T*. *brucei* MMS damage response. Kinetoplastids are unusual amongst single cell eukaryotes in encoding four Target of Rapamycin (TOR) PKs, which signal a wide range of cellular activities [58]. Two of the four *T*. *brucei* TOR PKs (TOR1 and TOR4) displayed MMS+/MMS- ratios <1 (0.75 and 0.74, respectively) and TOR4 was recovered in a kinome-specific MMS RIT-seq analysis (see below), despite being essential for growth [59], suggesting at least one arm of the expanded *T*. *brucei* TOR signalling network contributes to the MMS damage response.

Around 20% of genes in the MMS+/MMS- <0.5 gene set (‘unknown’ in Fig 2C) have annotated functions that have not been associated with the MMS damage response, many of which may be false positives, since they have roles in cellular processes that are unlikely to contribute to tackling MMS damage (e.g. cell motility). Others provide predicted functions that cannot be readily discounted (e.g. multiple peptidases and proteins implicated in cell division)[28]. Perhaps, given the wide range of functions recovered in DNA damage screens [60], some of these factors may yet prove to act in the *T*. *brucei* response to MMS-mediated DNA damage.

### New *T*. *brucei* damage response pathways revealed by RIT-seq

Enrichment of GO terms associated with DNA Pol and PK activities, as well as nuclear transport, suggested potentially unexplored *T*. *brucei* MMS damage response pathways (Fig 2C and 2D) that were not predicted by RNAi in *D*. *melanogaster* [29]. The clearest cohort of genes associated with nuclear and nucleocytoplasmic transport detected in the MMS+/MMS- <0.5 gene set were ras or RAB GTPases (Fig 2C), a class of enzymes that also contributes to chromosome segregation and cytokinesis in other eukaryotes [61]. In *S*. *cerevisiae*, mutants of nuclear pore complex factors are sensitised to MMS exposure [27], but only four of 20 annotated *T*. *brucei* nucleoporin proteins had MMS+/MMS- ratios of <1 (S1 Table)[62]. Thus, the putative *T*. *brucei* nuclear transport response to MMS damage is unclear from the available data. In contrast, analysis of DNA Pol activities was more revealing. We generated two plots: the MMS+/MMS- ratios of all genes that have been annotated as DNA Pols and all genes whose annotation implicates them in genome replication (Fig 3B and 3C; S2 Table). Together, these plots reveal that proteins providing core functions in genome replication do not act in the MMS damage response. For example, only one of the six subunits of the MCM2-7 replicative helicase [63, 64] and only two of the five subunits of the divergent Origin Recognition Complex [65] had an MMS+/MMS- ratio <1. Instead, putative accessory replication factors appear to play a crucial role in responding to MMS damage: translesion DNA Pols promote DNA replication across damage that cannot readily be repaired [66] and it is these enzymes that account for GO term enrichment of DNA Pol in the MMS+/MMS- <0.5 gene set. Only two translesion DNA Pols have been examined functionally in *T*. *brucei*, PrimPOL1 and PrimPOL2 [67]. Our RIT-seq screen indicated PrimPOL2 acts in the MMS damage response (Fig 3A), consistent with the protein accumulating at DNA damage lesions after MMS exposure [67]. However, the most notable translesion DNA Pol identified in our screen was DNA POLK (Kappa; Fig 3A), an enzyme found in multicopy in *T*. *brucei* but only in duplicate in *T*. *cruzi* [68], where one isoform localises to the kinetoplast and permits bypass of 8-oxo-guanine lesions (an oxidised base generated by MMS). The selection pressure that led to POLK expansion in *T*. *brucei* is unknown. A second putative MMS damage-response translesion DNA Pol is a putative homologue of the Rev3 component of DNA Pol zeta (POLZ)(Fig 3B and 3C), a multisubunit B family DNA Pol [69] that has not been examined in any kinetoplastid. A further gene (MMS+/MMS- ratio 0.63) encodes a putative subunit of Poly(A) Pol (Fig 3A), which may be of interest because RNA processing enzymes are emerging as playing direct and indirect roles in responding to DNA damage [70, 71]. In the broader class of replication-associated genes, the most prominent hit (MMS+/MMS- 0.29; Fig 3C) putatively encodes MCM8, a replicative helicase paralogue that acts with MCM9 to promote HR [72], which also has not been examined in kinetoplastids.

The above data implicate a range of DNA replication functions in the *T*. *brucei* response to MMS, consistent with the need to complete S phase after damage (28). To ask if wider genome-associated activities act in the *T*. *brucei* MMS damage response, we examined the MMS+/MMS- ratios of genes with annotated chromosome- (Fig 3D) and chromatin-associated (S2 Table) functions. Structural maintenance of chromosome (SMC) proteins play widespread roles in eukaryotic genome maintenance [73], though RNAi of neither the primary *T*. *brucei* cohesin (SMC1 and SMC3) nor condensin (SMC2 and SMC4) subunits resulted in loss of reads after MMS exposure (Fig 3C), suggesting no roles in damage repair. This is perhaps surprising, given that *T*. *brucei* homologues of SMC5 or SMC6 (which provide repair functions amongst eukaryotic SMC complexes)[74] have not been identified [75]. Perhaps SMC5/6 functions are assumed by the two putative nuclear *T*. *brucei* Topoisomerase II isoforms [76–78](S2 Table). A further *T*. *brucei* topoisomerase, Top3α, displayed an MMS+/MMS- ratio of 0.87 (S2 Table), consistent with sensitivity of null mutants to other forms of damage [79]. It has long been known that eukaryotic telomeres present a paradox, in being DNA ends that do not elicit a damage response [80]. Four *T*. *brucei* telomere-associated factors, including KU70 and KU80, each displayed MMS+/MMS- ratios <1 (Fig 3C), in keeping with predictions that such factors impede DNA damage signalling and inappropriate repair. Unlike TRF [81] or RAP1 [82], KU null mutants have been described and are viable [83, 84] but lack sensitivity to DNA damaging agents, consistent with the apparent lack of KU-mediated NHEJ in kinetoplastids [5–9]. MMS-sensitivity after RNAi suggests the primary role of KU is telomere-related and complete loss of KU may cause adaptation to cope with telomere attrition [83, 84], such as by alternative lengthening of telomeres[85]. Amongst factors implicated in *T*. *brucei* chromatin (S2 Table), 21 genes encoding potential chromatin modifying factors displayed MMS+/MMS- ratios <1, three of which were putative SIR2-related factors: null mutants of the nuclear factor, SIR2rp1, shows sensitivity to MMS [86]. Furthermore, a putative bromodomain-containing factor (BDF2) was identified that has not been examined functionally in *T*. *brucei* but whose expression increases after UV damage in *T*. *cruzi* [87]. The number of, to date, unexplored factors suggests wider roles for chromatin in the response to damage, either by directly effecting repair or by altering transcription or replication [88]. One group of factors was putative arginine methyltransferases (PRMTs), of which three out of four annotated genes displayed MMS+/MMS- ratios <1 (S2 Table). PRMT6 displayed the lowest ratio (0.56) and has been previously implicated in *T*. *brucei* cytokinesis [89], while PRMT1 in *Toxoplasma gondii* influences chromosome replication [90], suggesting some of these factors act in genome maintenance.

### Genome-wide RIT-seq reveals multiple novel protein kinases that act in the *T*. *brucei* MMS damage response

**Fig 4:**
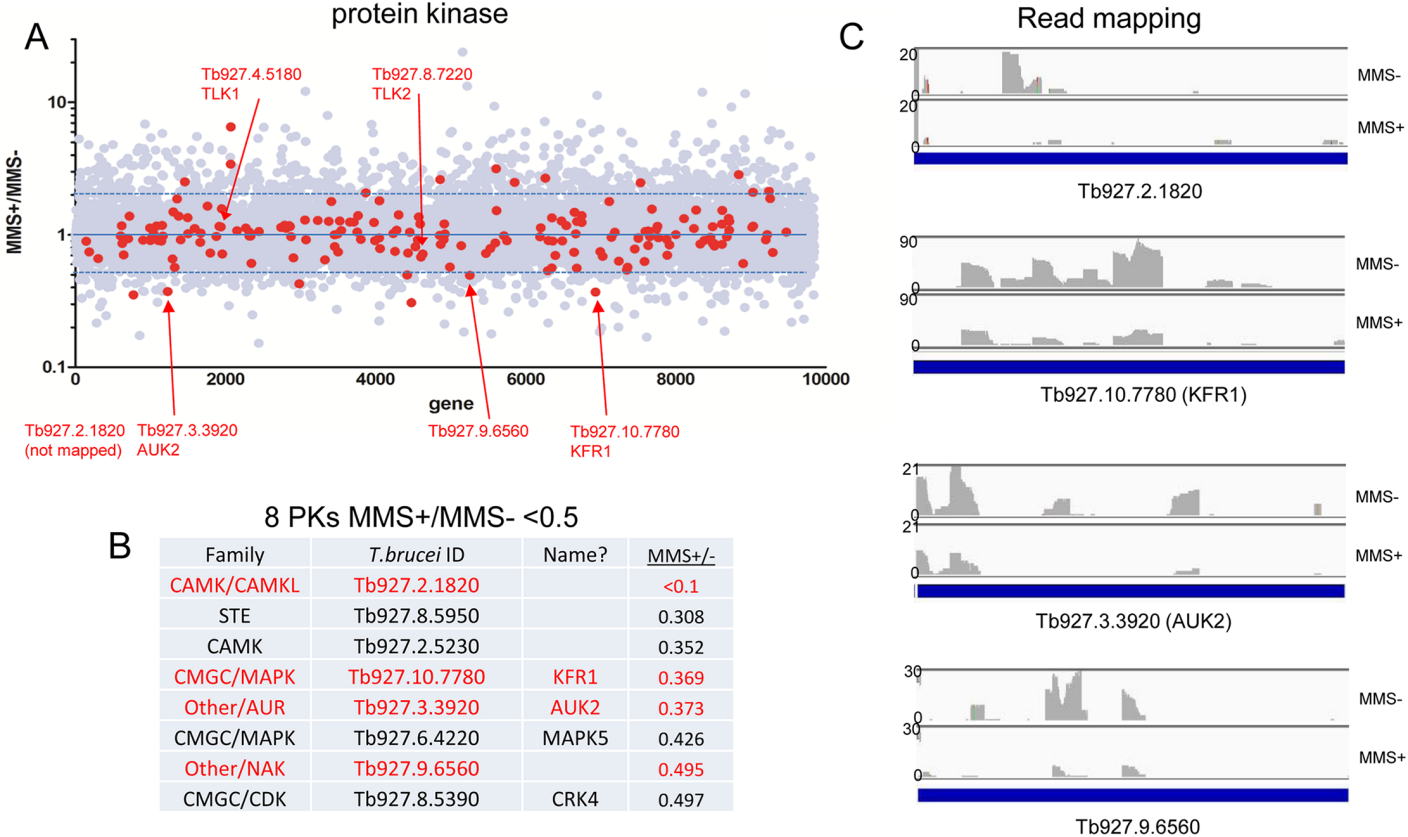
Damage response protein kinases predicted by whole-genome MMS RIT-seq. **A.** Scatter plot of MMS+/MMS- read depth ratios for all *T*. *brucei* genes (grey dots). Protein kinase (PK) genes are highlighted in red and individual genes are further identified (arrows) by gene IDs and names, if known (further details in S2 Table). **B.** Nine PK genes, including PK family and name, present in the MMS+/MMS- <0.5 gene set are listed. **(C** displays read mapping profiles for selected PKs (red) after RNAi and growth with (MMS+) or without (MMS-) 0.0003% MMS (see Fig 5 and S5 and S6 Figs for more detail).

**Fig 5:**
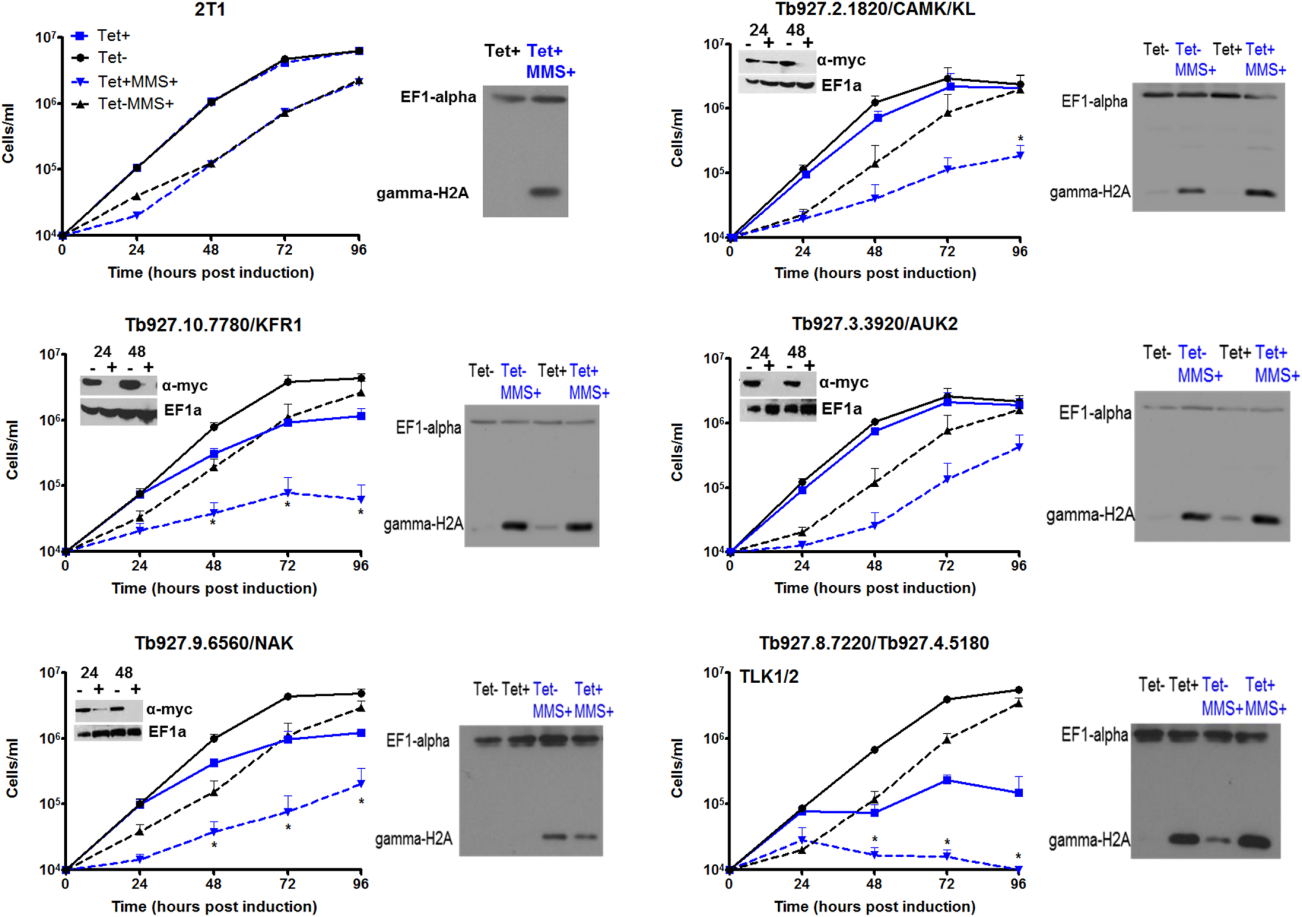
*In vitro* growth of putative MMS damage response protein kinases identified by genome-wide MMS RIT-seq. Individual tetracycline (Tet) inducible RNAi cell lines were generated for five PK genes (identified by gene ID and name, if known) and their growth assessed by counting parasite density every 24 hrs for 96 hrs. Growth was assessed in the absence (-) and presence (+) of MMS (0.0003% v/v) and with (+) or without (-) Tet RNAi induction. The same analysis is shown for parental 2T1 cells, which do not induce gene-specific RNAi. Each data point displays the mean cell density from three independent biological replicates error bars represent SEM. Significant differences between the means of the Tet-, MMS+ sample relative to the Tet+, MMS+ were calculated using a Mann Whitney U test; (*) = p<0.05, which was considered significant. Within each graph, protein loss for 12myc-tagged PKs was tested by western blot analysis on whole cell extracts using anti-myc antiserum. Cells were harvested after 24 and 48 hrs growth with (+) or without RNAi induction by addition of Tet. Anti-EF1-α was used as a loading control. Beside each graph γH2A expression levels after RNAi against the PKs, with (MMS+) or without exposure to MMS (MMS-), are shown in western blots. Cells were induced (with 1 μg.ml^-1^ tetracycline; Tet+) or left uninduced (Tet-) for 24 hrs. At 24 hrs, MMS (to a concentration of 0.0003% [v/v]) was added to an induced and an uninduced culture. Whole cell lysates were collected, separated on gels, blotted and γH2A was detected using anti-γH2A antiserum (anti-EF1α was used as a loading control). Gene IDs and names are provided for the PKs genes analysed; 2T1 is the parental cells, where Tet does not induce dsRNA.

PKs were amongst the largest gene family that displayed GO enrichment (Fig 2D, S3 Table), with eight genes belonging to six functional PK families (Fig 4) in the MMS+/MMS- <0.5 gene set, as well as a PK regulator (S3 Table). None of these eight PKs has been predicted to provide damage response functions and so we tested the RIT-seq prediction of this novel gene cohort. We first evaluated the sequence mapping for each gene and found consistently lower reads for six of the eight PK genes (Fig 4C, S4A Fig) in the Tet+, MMS+ cells compared with the Tet+, MMS-; for the two other genes (Tb927.2.5230 and Tb927.6.4220) the average RIT-seq ratios (Fig 4B) masked variation in read depth between the replicates (S4B Fig) and so these PKs were not tested further. For the six remaining PKs, BSF cells carrying a single Tet-inducible RNAi target for each PK gene [91] were used to monitor growth before and after RNAi induction in the presence or absence of 0.0003% MMS (Fig 5, S4A Fig). For comparison, growth analysis was also conducted with the parental 2T1 cell line (which does not induce dsRNA targeting any gene)[92]. We also examined the *T*. *brucei* homologues of tousled-like kinase (TLKs). Though *T*. *brucei* TLK1 and TLK2 did not display MMS+/MMS- ratios <0.5 (1.15 and 0.68, respectively), metazoan TLKs contribute to DNA damage signalling and recovery [93, 94] and conserved interactions have been described for the *T*. *brucei* TLKs (phosphorylation by an aurora kinase, and phosphorylation of histone H3 and anti-silencing factor homologues)[95]. As expected, Tet addition had no effect on growth of 2T1 cells, and addition of MMS slowed growth (Fig 5). Induction of RNAi that targeted both TLK1 and TLK2 caused slowing of growth, comparable to that seen after TLK1-specific RNAi in PCF cells [95], and the growth reduction caused by MMS was exacerbated (Fig 5), indicating loss of one or both TLKs causes increased MMS sensitivity. For four PKs (Fig 5) we translationally fused the endogenous gene with 12 copies of the myc epitope in the cognate RNAi cell and, in all cases, loss of tagged protein was seen 24 or 48 hrs after RNAi induction, with modest slowing of growth in two cases (Tb927.10.7780, KFR1; Tb927.9.6560) and little change in the others (Tb927.3.3920, AUK2; Tb927.2.1820)(Fig 5). For each of these PKs, addition of MMS after RNAi resulted in slower growth than in MMS-treated uninduced cells or RNAi-induced untreated cells, indicating loss of each PK sensitises BSF *T*. *brucei* to alkylation damage, consistent with the RIT-seq screen. Preliminary growth analysis of the final two PKs, Tb927.8.5890 and Tb927.8.5390 (CRK4), without evaluation of RNA or protein levels (S4A Fig), provided no clear evidence for increased MMS sensitivity after Tet addition. It is possible these genes are false positives, but kinome RIT-seq (below) provides support for the whole-genome RIT-seq analysis of CRK4.

To ask if the four novel PKs and TLK1/2 act in genotoxic stress signalling, we evaluated levels of γH2A, which were low in untreated 2T1 cells but increased substantially after 48 hrs growth in 0.0003% MMS (Fig 5). TLK1/2 RNAi resulted in elevated γH2A levels in the absence of MMS, indicating that loss of this PK resulted in accumulation of nuclear genome damage. A similar but lesser increase in γH2A levels was seen after RNAi without MMS for KFR1 and AUK2. The absence of a detectable increase in γH2A after RNAi against Tb927.9.6560, which causes a notable growth defect (Fig 5), suggests H2A modification is not merely a result of defective BSF cell replication. Levels of γH2A after MMS exposure and RNAi were never lower than that seen in uninduced cells treated with MMS, and showed limited evidence for further increases, indicating that none of these PKs strongly influence the phosphorylation or dephosphorylation of H2A.

To ask if the PKs have roles in regulating cell cycle progression, such as checkpoint signalling after damage, DNA was stained with DAPI in fixed cells from each RNAi cell line 24 and 48 hrs after RNAi, with or without exposure to 0.0003% MMS (S5 Fig). Visualisation of the nuclear (N) and kinetoplast (K) DNA permits the approximate cell cycle stage of individual cells in a population to be assessed [96]. Only for TLK1/2 did RNAi without MMS cause a pronounced change in cell cycle distribution (S5 Fig); this change differed from the effect described following RNAi of TLK1 in PCF cells [95] in that accumulation of 1N2K (S/G2) cells was not seen and, instead, cells emerged with aberrant N and K configurations, including 0N1K ‘zoids’. For all of the PK cell lines, MMS treatment without induction of RNAi did not result in a detectable accumulation of cells in a specific cell cycle stage, but instead reduced numbers of 1N1K (G1/S), 1N2K and 2N2K (post-M) cells were seen with an associated accumulation of cells with aberrant DNA content. Perhaps surprisingly, these data suggest BSF *T*. *brucei* cells continue to undergo cell division and DNA replication after MMS exposure, meaning they do not enact a clear checkpoint after treatment and mis-segregate their damaged genomes. Nonetheless, RNAi of each PK in the presence of MMS resulted in greater numbers of aberrant cells, consistent with increased MMS sensitivity.

### A kinome-focused MMS RIT-seq screen reveals further damage response kinases

**Fig 6:**
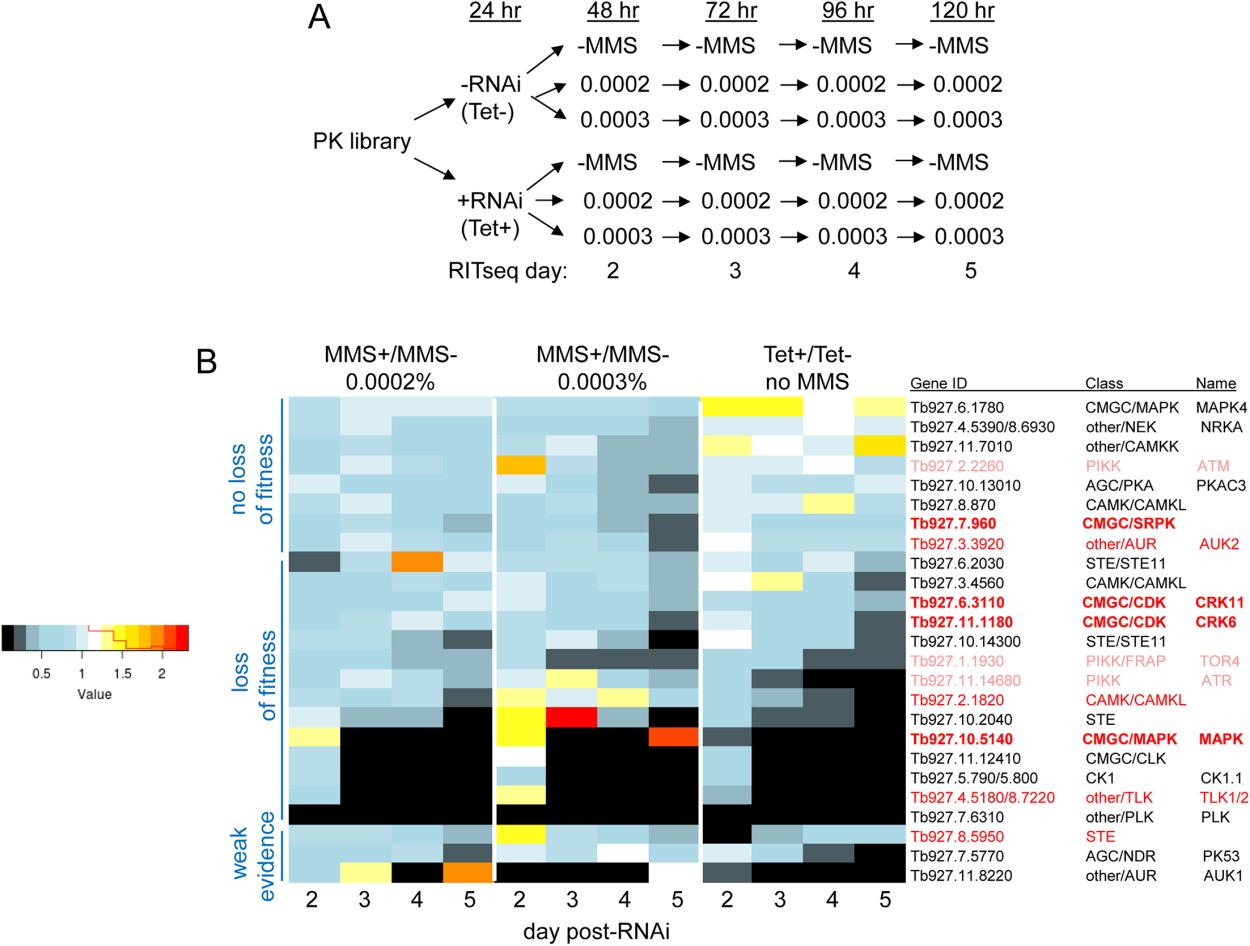
A kinome-focused MMS RIT-seq screen. **A.** A pool (library) of BSF *T*. *brucei* cells was generated allowing parallel tetacycline (Tet) induction of RNAi against all protein kinase (PK) genes. The pool was split, RNAi initiated in one culture (Tet+) and the other grown without RNAi (Tet-). After 24 hrs both cultures were further split and grown for four days in the presence of either 0.0002% or 0.0003% MMS, or without addition of damaging agent (MMS-). DNA was prepared from the populations on days 2, 3, 4 and 5 and PCR used to recover the PK-specific RNAi targets. PCR products were sequenced and mapped to the PK genes, determining read abundance in the different conditions and at different times. **B.** A heatmap of ratios of reads in the different populations shown in A are detailed for putative MMS damage response PK genes, which are identified by gene ID, PK class and name (if known); all PK genes are provided in S4 Table. For each gene MMS+/MMS- ratios are shown at each of the 4 days examined and at both MMS concentrations; to evaluate the importance of the genes for cell survival, ratios of RNAi target reads from the Tet+ cells relative to the Tet- cells, without addition of MMS, are shown at the same time points. Genes highlighted in bold red were not seen in the genome-wide MMS RIT-seq but were validated by targeted RNAi (Fig 7); genes in red are common between the two MMS RIT-seq screens; and genes in pink have predicted roles in MMS damage repair in other eukaryotes.

The whole genome MMS RIT-seq strategy we adopted is limited for two main reasons. First, we sampled at only one time point (5 days post-RNAi induction), meaning essential genes may be missed. Second, RNAi target number per gene is variable, meaning mapping coverage may be limited in some cases, such as for small genes. To address these limitations for PKs, we took advantage of the availability of a kinome-wide library of BSF *T*. *brucei* cells [91], which allows Tet-induced RNAi using a single, defined RNAi target for each putative PK. 177 clonal RNAi cell lines, targeting 183 PKs, were pooled to allow kinome-wide MMS RIT-seq. The pooled cells were first inoculated at a density of 1 x 10^5^ cells.ml^-1^ and grown for 24 hrs without or with addition of Tet, providing a control population and an RNAi-induced population, respectively (Fig 6A). The two populations were then each split into three and grown without addition of MMS, or with the addition of 0.0002% or 0.0003% MMS. The six resulting populations were all grown for a further four days and genomic DNA prepared each day. To determine the abundance of PK-targeting cells in the populations and at the increasing time points, limited cycle PCR was performed from the DNA preparations using primers that amplify each PK RNAi target. The PCR reactions were then sequenced and mapped to a minimal genome, equivalent to the whole-genome RIT-seq but here limited to the PK RNAi targets. Normalised MMS+/MMS- ratios for each day and at both MMS concentrations are shown for every PK gene in S4 Table, while genes that show, after RNAi, reduced reads in the presence of MMS are highlighted in Fig 6B.

The advantage of the kinome RIT-seq was most apparent in the ability to follow changes in PK gene levels with time. As shown in Fig 6B, 22 genes followed a pattern of decreasing MMS+/MMS- ratios from days two to five, and greater read losses at 0.0003% MMS compared with 0.0002%: eight genes (‘no loss of fitness’ in Fig 6B) registered no significant fitness cost after RNAi, as judged by unchanged read levels in the Tet+, MMS- control samples; for 14 genes (‘loss of fitness’ in Fig 6B), reads diminished with time in the same controls, indicating loss of fitness after RNAi. Three further genes (‘weak evidence’ in Fig 6B) showed some evidence for increased sensitivity to MMS after RNAi, but with less clear time dependence. The kinome-focused MMS RIT-seq revealed two things: confirmation of the whole-genome RIT-seq, and an expanded repertoire of MMS damage response PKs.

Amongst the eight PKs with MMS+/MMS- ratios <0.5 in the whole genome RIT-seq, three were identified in the kinome screen with MMS+/MMS- ratios of <0.5 (Fig 6B). Two (AUK2 and Tb927.2.1820) were validated by independent RNAi (Fig 5), while the third (Tb927.8.5950) was only subject to preliminary growth analysis (S4A Fig), but its presence in both screens strengthens the suggestion it is an MMS damage response PK. Two further whole-genome RIT-seq PKs (Tb927.2.5230 (CRK4), Tb927.6.4220) were not pursued due to inconsistent mapping (S4B Fig), while preliminary RNAi of one other (Tb927.8.5390; S4A Fig) was not supportive of an MMS damage response function. Intriguingly, though each of these PKs did not display MMS+/MMS- ratios <0.5 in the kinome screen, all displayed modest loss of reads in the MMS-treated cells relative to the untreated, an effect that increased with time and at the higher MMS level (S4 Table). The kinome RIT-seq also clearly revealed TLK1/2 as showing MMS-specific read losses after RNAi. Overall, therefore, seven of nine PKs considered to this point showed good correspondence between the whole-genome and kinome-focused RIT-seq data, though only three have been validated by targeted analyses. Two whole-genome RIT-seq PKs, KFR1 and Tb927.9.6560, showed no evidence for MMS-specific read losses in the kinome RIT-seq (S4 Table). The basis for this discord is unclear, since independent RNAi validated both PKs as MMS damage response factors (Fig 5).

**Fig 7:**
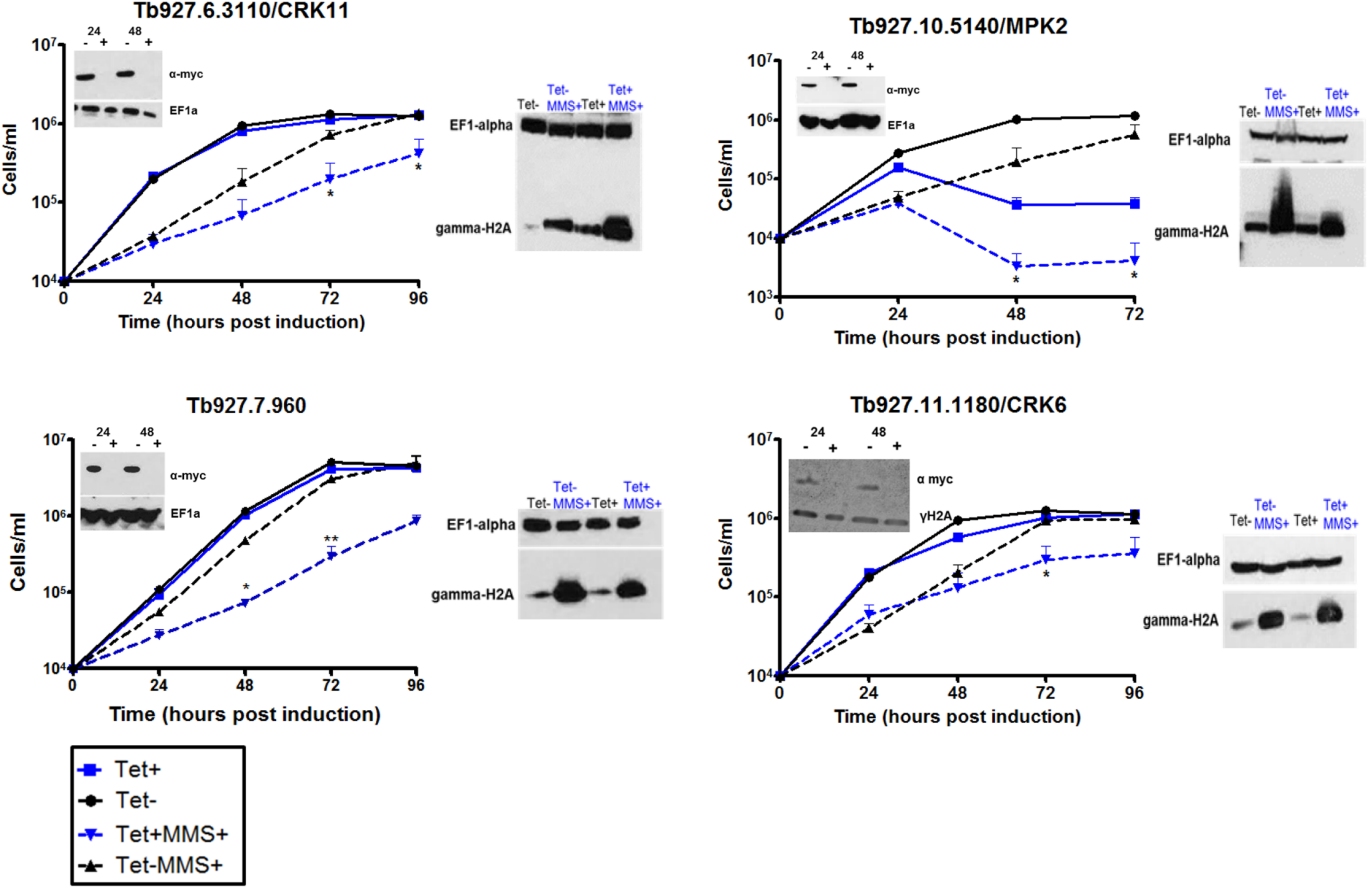
*In vitro* growth of putative MMS damage response protein kinases identified by kinome-focused MMS RIT-seq. Individual RNAi cell lines were generated for four PK genes (identified by gene ID and name, if known) and their growth assessed by counting parasite density every 24 hrs for 96 hrs, as described in Fig 5. Protein loss was tested by western blot analysis on whole cell extracts, as was γH2A expression level after RNAi (Tet+) against the PK, with (MMS+) or without exposure to MMS (MMS-); experimental details are as described in Fig 5.

Within the expanded repertoire of putative MMS damage response PKs predicted by the kinome RIT-seq (Fig 6B), the putative *T*. *brucei* homologues of ATM, ATR and TOR4 were recovered (the latter two showing evidence for loss of fitness, consistent with growth analysis after targeted RNAi)[59, 91]. None of these PKs have been examined for a role in *T*. *brucei* DNA repair or the MMS damage response, but such functions are consistent with work in other eukaryotes [29]. To test the wider kinome RIT-seq predictions, we performed targeted RNAi for seven PKs (Fig 7, S6 Fig). Preliminary growth curves suggested RNAi against only two PKs (S6B Fig) did not result in increased MMS sensitivity, whereas growth of the five others (Fig 7, S6A Fig), spanning four functional PK classes, was slower after RNAi in the presence of 0.0003% MMS relative to growth in the absence of damage or in the presence of MMS without RNAi. Addition of a 12myc tag to four of the PKs demonstrated loss of protein after RNAi (Fig 7), though we have so far been unable to similarly tag one PK (Tb927.11.7010)(S6A Fig), which we therefore did not analyse further. Only for Tb927.10.5410 (MPK2) did slowed growth following RNAi without MMS clearly mirror the RIT-seq predicted loss of fitness (Fig 7), resulting in a pronounced accumulation of aberrant cells that was not seen following RNAi against the three other PKs (S7 Fig). To ask if the PKs might act in genome maintenance, we examined γH2A levels (Fig 7). RNAi against none of the PKs abrogated the MMS-dependent increase in γH2A signal, indicating none mediate this phosphorylation. However, for Tb927.6.3110 (CRK11)[97], γH2A levels increased after RNAi without MMS, despite the lack of a growth defect, and the signal was higher after RNAi induction and MMS treatment than in MMS-treated cells without RNAi induction, suggesting loss of this PK causes accumulation of nuclear DNA damage (Fig 7). No such effects were seen after RNAi against Tb927.10.5410, which resulted in impaired growth (Fig 7), nor Tb927.11.1180 (CRK6)[98], where some accumulation of aberrant cells was seen (S7 Fig). Finally, though the RIT-seq and growth analysis suggest Tb927.7.690 (which encodes a predicted CMGC/SRPK class PK) is non-essential, further RNAi data indicate an important role in *T*. *brucei* survival in mice [99].

### Ablation of AUK2 sensitises BSF *T*. *brucei* to genotoxic stress

Tb927.3.3920 encodes AUK2, one of three predicted *T*. *brucei* aurora kinases (AUKs)[100]. The presence of three AUKs in a single-celled eukaryote is unusual, since whereas mammals have three (AUKA, AUKB and AUKC), yeast and *Dictyostelium discoideum* have a single AUK. Mammalian AUKA and AUKB have important but distinct roles in mitosis and cytokinesis by monitoring and contributing to centrosome function, microtubule attachment to the centromere and chromosome segregation, while AUKC appears to act during meiosis [101]. Functional studies in *T*. *brucei* have focused on AUK1, which is essential, provides AUKB-like functions [102, 103] and is considered a drug target [104, 105], building on anti-cancer compounds that target AUKs. Why kinetoplastids express two further AUKs, and whether they might also be targets for chemotherapy, is unclear.

**Fig 8:**
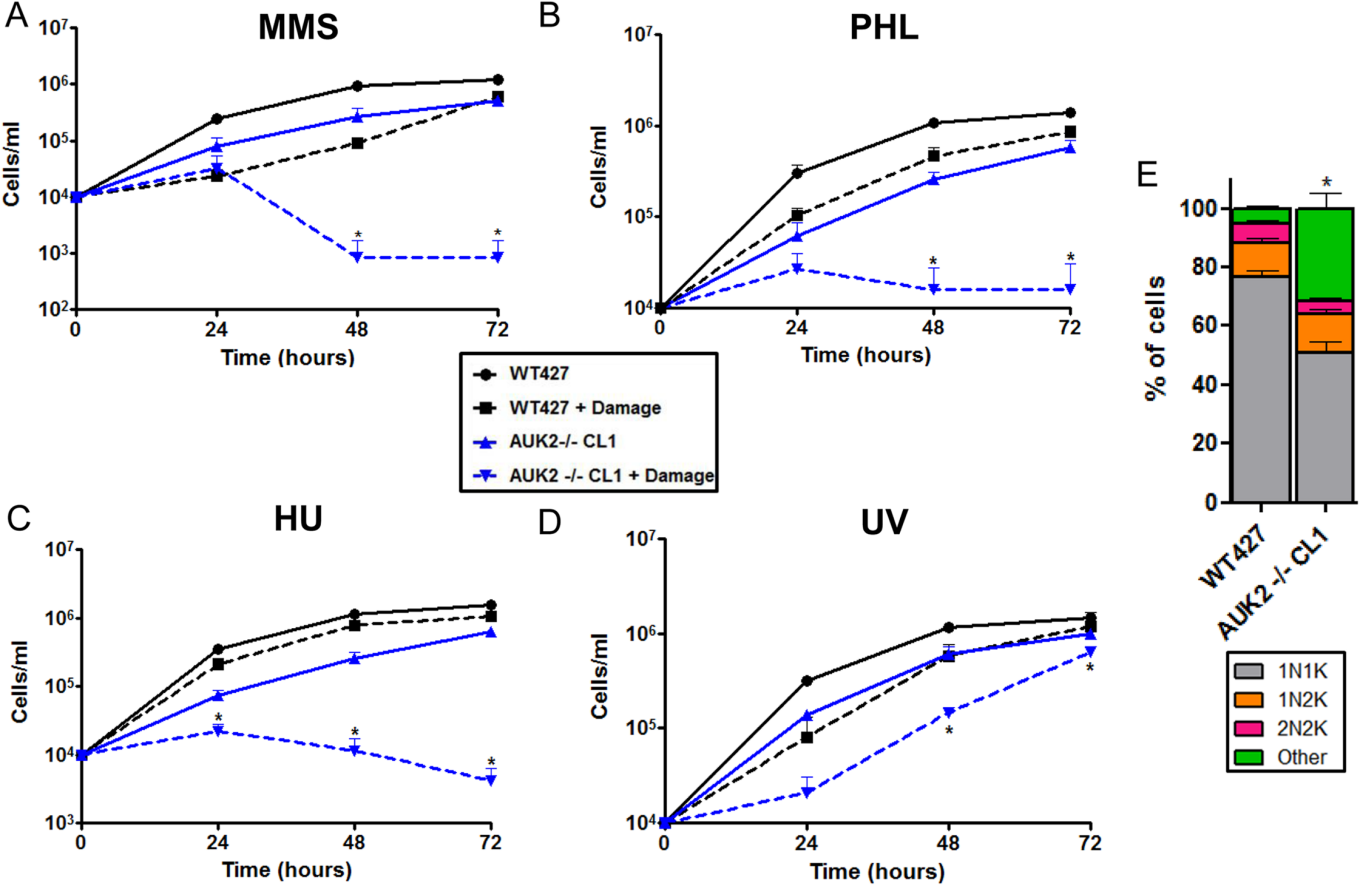
Loss of AUK2 sensitises *T*. *brucei* to DNA damaging agents and results in altered cell cycle progression. **A-D** Growth curves of one *auk2* -/- null mutant clone (CL1) compared with wildtype (WT427) cells; cell density was monitored every 24 hrs for 72 hrs in the presence (+) and absence of MMS (0.0003%), phleomycin (PHL; 0.1 μg ml^-1^), hydroxyurea (HU; 0.6 mM; C) or after exposure to UV (1500 J/m^2^). All graphs show mean density from three experiments; error bars denote SEM. Significant differences are shown by * (P<0.05; Mann Whitney U test). **E.** Cell cycle analysis of *auk2* -/- mutants compared with WT cells. Cells were harvested, fixed and stained with DAPI for visualisation of the kinetoplast (k) and the nucleus (n). >200 cells were counted from three independent replicates of each cell type, and the n-k configuration of individual cells expressed as a percentage of the total population. Cells that did not show any of the expected N-K configurations (1N1K, 1N2K or 2N2K) were categorised as ‘other’. Error bars represent SEM. * P<0.05 (Mann Whitney U test; comparison between WT other cells and AUK2 -/- CL1 other cells).

RNAi of AUK2 had little effect on BSF *T*. *brucei* growth (Fig 5, S4 Table), suggesting the PK is not essential *in vitro*. To test this, null mutants were generated in BSF cells by replacing the two allelic ORFs with antibiotic resistance markers (S8A–S8C Fig). Though viable, *auk2* null (-/-) mutants displayed significantly impaired growth relative to wild type (WT) cells *in vitro* (Fig 8). Furthermore, a significant increase (~6 fold) in cells with aberrant N-K ratios was seen in the -/- mutants relative to WT (Fig 8E) or heterozygous cells (+/-)(S8D Fig), with a range of abnormal DNA configurations observed (S8E Fig). Growth of *auk2-/-* mutants was significantly more impaired than WT cells in the presence of MMS (Fig 8A), consistent with the *AUK2* RIT-seq and RNAi data. Indeed, MMS sensitivity after AUK2 loss appears to reflect a wider role for this PK in response to genotoxic damage, since the *auk2-*/- mutants also grew more slowly than WT cells in the presence of phleomycin or hydroxyurea, and after exposure to UV (Fig 8B–8D, S9 Fig).

**Fig 9:**
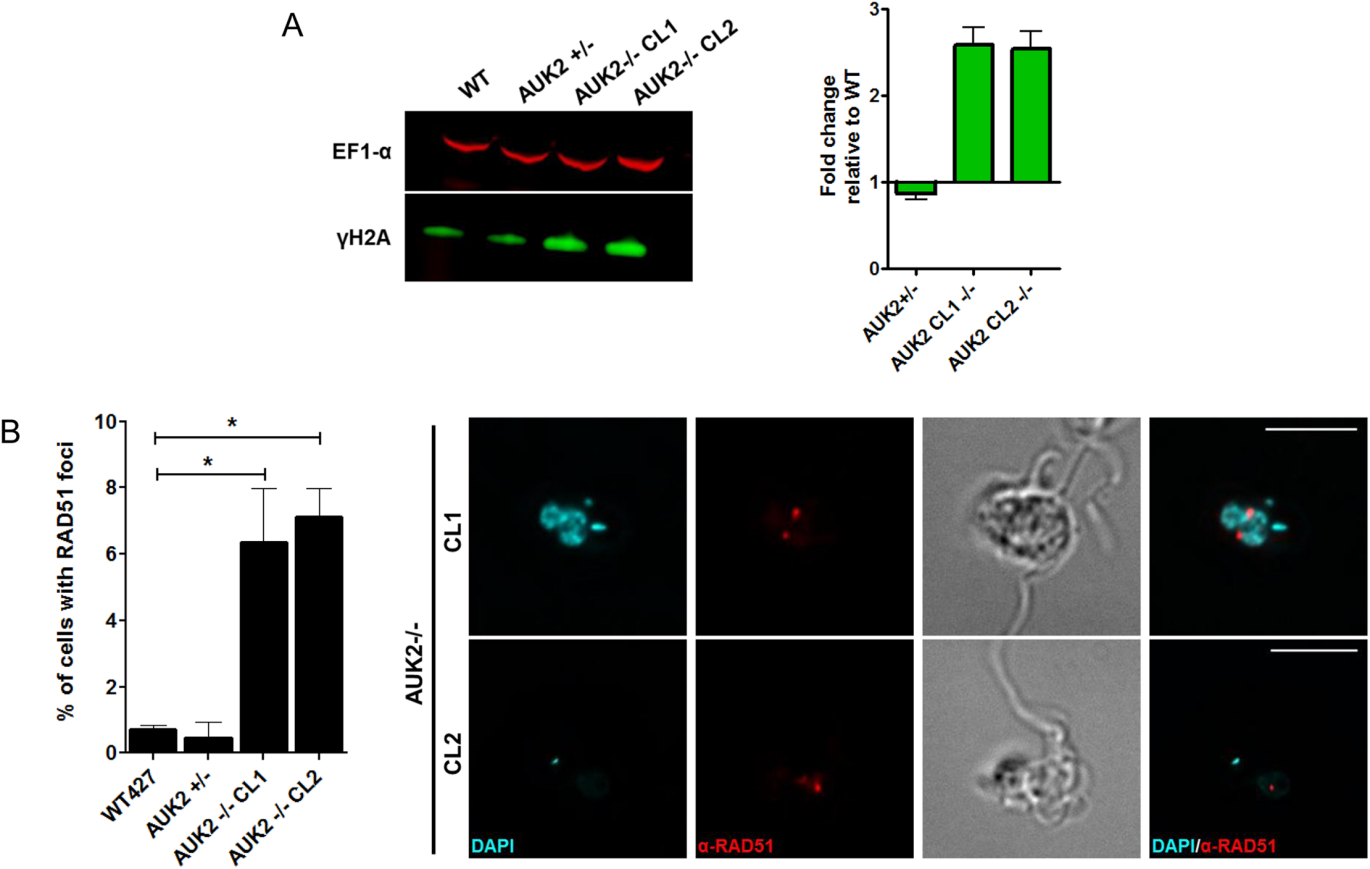
Loss of AUK2 results in nuclear DNA damage. **A.** Western blot analysis of γH2A in two *auk2* -/- mutants clones (CL1 and CL2) relative to an *AUK*+/- heterozygous mutant and wild type cells (WT427). Whole cell lysates were probed with anti-γH2A (below) and anti-EF1α (above; loading control) antisera. The graph shows levels of γH2A after normalisation by EF1α: γH2A levels in WT cells were set at 1 and fold change in the mutants relative to WT is shown. Data points represent means and SEM (n = 3). **B.** Immunofluorescence (IF) of RAD51 foci formation. Cells were harvested, fixed and RAD51 localised with anti-RAD51 antiserum. Representative IF images of *auk2*-/- mutants are shown in which DAPI stained DNA is in blue and RAD51 in red (cell morphology is shown by differential contrast imaging); the scale bar = 10 μm. The graph shows the percentage of WT cells with detectable RAD51 foci compared with *AUK2*+/- mutants and two *auk2*-/- clones. Cells with RAD51 foci are represented as a percentage of the total population of cells counted (n >200). Data points represent the mean from three independent experiments; errors bars show SEM. * denotes a significant difference from WT (P<0.05, Mann Whitney U test).

To ask if AUK2 acts in the *T*. *brucei* DDR, levels of γH2A were assessed by western blot, revealing a 2.5-fold increased expression in two null mutant clones relative to WT (Fig 9A, S10A Fig); indeed, immunofluorescence imaging indicted greater numbers of -/- cells than WT displayed nuclear γH2A signal (S10B Fig). To explore this increased endogenous damage further, indirect immunofluorescence was performed to examine localisation of RAD51, a factor that binds ssDNA at a DNA break, which can be observed as localisation to discrete sub-nuclear foci. ~1% of WT cells displayed RAD51 foci (Fig 9B), consistent with previous reports [47], but this basal level increased to 6–7% in the *auk2*-/- mutants. Together, these data show loss of AUK2 affects integrity of the *T*. *brucei* nuclear genome, impedes survival following exposure to a range of genotoxic agents and impedes completion of the BSF cell cycle.

### AUK2 provides nuclear maintenance functions

**Fig 10:**
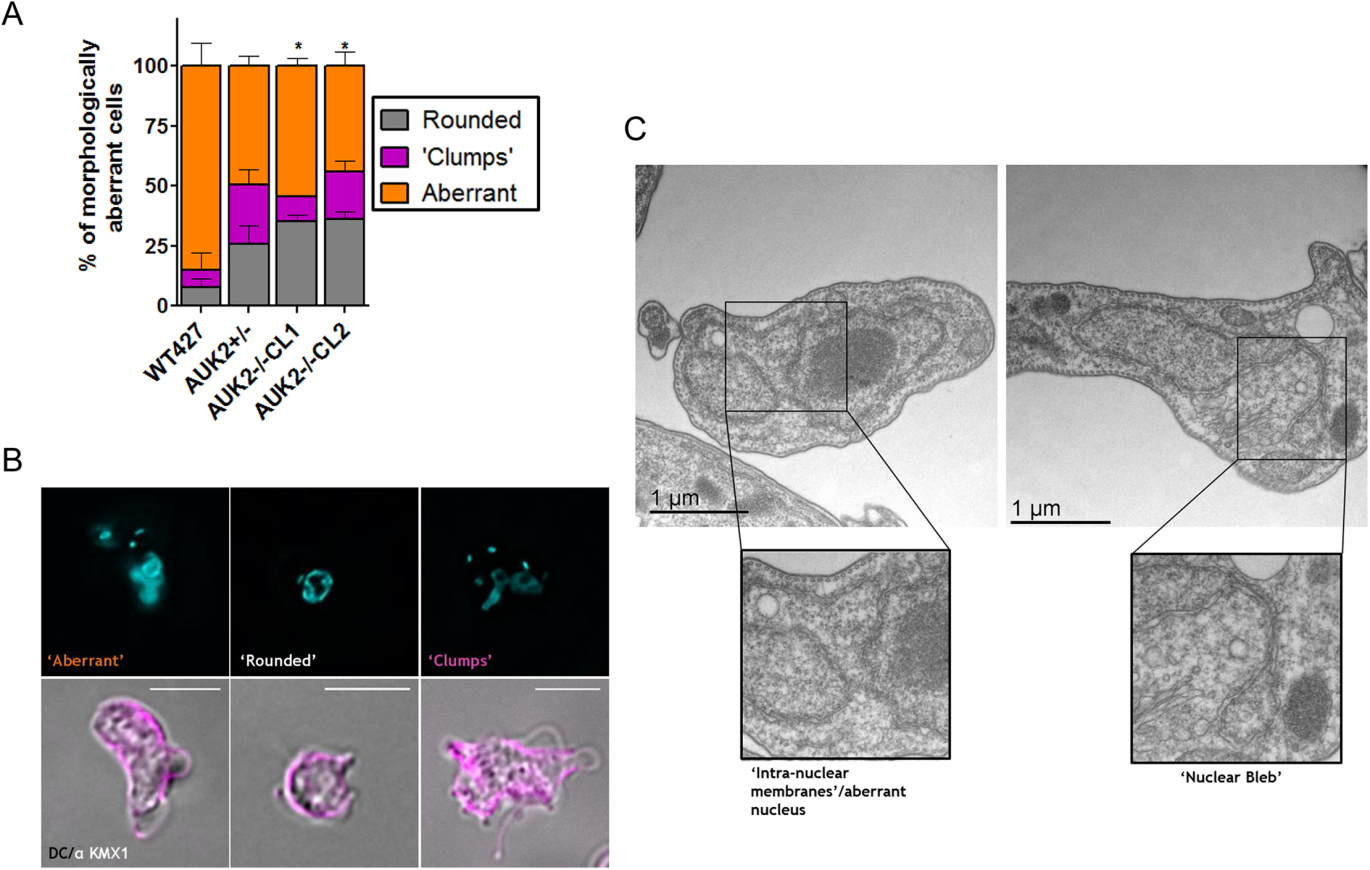
Loss of AUK2 results in aberrant cell and nuclear morphology. **A.** Wild type (WT427), *AUK2*+/- and *auk2*-/- cells (clones CL1 and CL2) with morphology that deviated from the typical BSF cell shape were classified into three categories: rounded, clumps or aberrant. Each category is shown as a percentage of the total number of cells with morphological defects; >200 cells were counted for this analysis, which was conducted in triplicate. Error bars represent SEM, and * denotes a significant difference (p<0.005; Mann Whitney U test) in the percentage of rounded cells seen in WT *T*. *brucei*. **B.** Representative images of rounded, clumped or aberrant *auk2*-/-cells; in each case, the upper image shows DNA stained with DAPI (blue), while the lower image shows a merge of differential contrast and staining with anti-KMX-1 antiserum (to visualise βtubulin; magenta). Scale bar = 5μm. **C.** Representative examples of nuclei in *auk2*-/- mutants visualised by transmission electron microscopy. Boxes show higher magnifications of an unusual arrangement of nuclear membranes (lower left), or a nuclear ‘bleb’ (lower right). Scale bar sizes are indicated.

To scrutinise AUK2 function further, cell and nuclear morphology of the *auk2*-/- mutants was examined. The cell body and the mitotic spindle in fixed WT and mutant cells were visualised by staining with anti-tubulin KMX-1 antiserum [106], and the N- and KDNA were stained with DAPI. Only ~4% of WT cells deviated from the typical *T*. *brucei* BSF shape, a proportion that increased to ~35% of the *auk2*-/- population, a ~9-fold increase that closely mirrored the increased numbers of null mutants with aberrant DNA content (Fig 8E). The predominant defect seen in WT cells was an enlarged, unclassifiable (‘aberrant’) cell morphology (~85% of aberrant cells)(Fig 10A). In contrast, ~25% of the aberrant *auk2*-/- cells displayed a characteristic ‘rounded’ morphology (Fig 10A and 10B), akin to defects reported following AUK1 RNAi silencing [103]. Increased levels of nuclear defects were also observed in the *auk2*-/- mutants. Electron microscopy (Fig 10C) revealed mutants with aberrant nuclear membrane organisation, including the presence of nuclear ‘blebs’ (which were seen in ~20% of *auk2*-/- mutants, a ~10-fold increase relative to WT; S11A Fig). Furthermore, the number of 1N2K cells with a detectable mitotic spindle was reduced by ~50% in the *auk2*-/- cells relative to WT (S11B Fig). Together, these phenotypes suggest loss of AUK2 results in impaired nuclear architecture and genome division, perhaps because of failure to enact appropriate damage checkpoints from G2 to cytokinesis.

**Fig 11:**
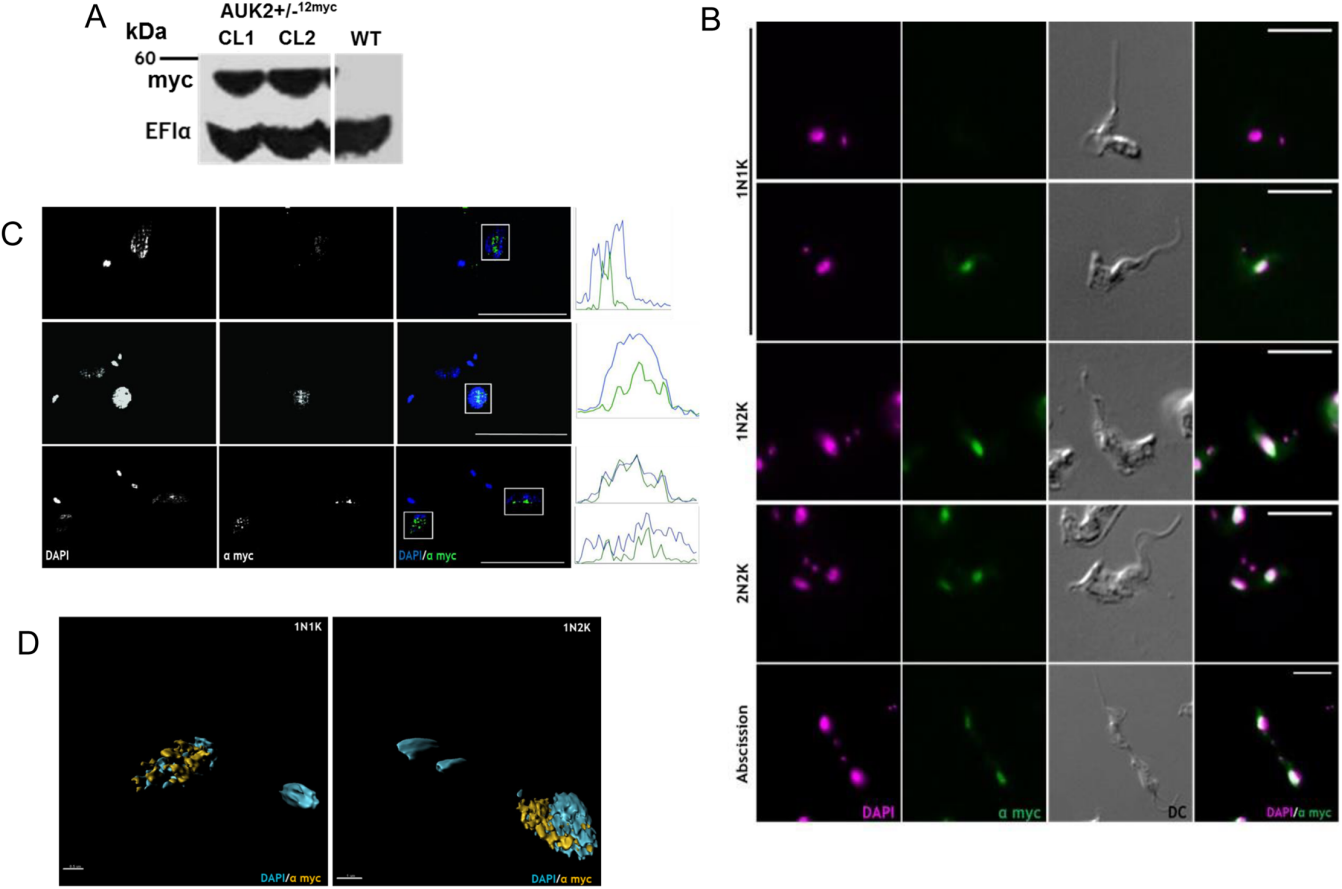
AUK2 displays dynamic nuclear localisation. **A.** Western blot of whole cell extracts from wild type (WT) *T*. *brucei* and from two clones in which the *AUK2* ORF has been C-terminally fused to a tag encoding 12 myc epitopes (*AUK2*+/-::12myc). The blot was probed with anti-myc and anti-EF1α antiserum (as a loading control); a size marker is shown. **B.** Representative images of *AUK2*+/-::12myc cells from each cell cycle stage (denoted by N-K ratio). Anti-myc antiserum was used to visualise myc tagged AUK2 (green) and nDNA and kDNA were stained with DAPI (magenta); DC imaging shows cell shape; scale bars = 5 μm. **C.** Super resolution images of AUK2-12myc localisation. Only in the merged images are DAPI (blue) and anti-myc signals (green) shown in colour. Graphs show fluorescence intensity (arbitrary units; AU) over distance plotted for both the DAPI (blue) and anti-myc (green) signals. The white box represents the area from which the fluorescence intensity was measured; scale bar = 5 μm. **D.** 3D reconstruction of AUK2-12myc localisation in a 1N1K or 1N2K cell.

To localise AUK2, 12 copies of the myc epitope were fused to the C-terminus of the protein by targeting the intact allele in *AUK2*+/- cells (Fig 11A). Unaltered growth of the resulting *AUK2*+/-::12myc cells relative to WT or *AUK2*+/- cells suggested expression of the tagged protein did not compromise function (S12A Fig). Indirect immunofluorescence with anti-myc antiserum revealed an exclusively nuclear signal (Fig 11B), though in ~10% of 1N1K cells no staining could be detected (S12B Fig). Structure illuminated super-resolution microscopy (Fig 11C) and 3D modelling (Fig 11D) revealed that AUK2-12myc localisation or expression is dynamic, with puncta seen throughout the nucleus in 1N1K cells and the signal relocalising to the centre of the nucleus in 1N2K cells. Consistent with dynamism, structure illumination microscopy could not resolve any localisation in 2N2K cells (S12D Fig) and myc signal varied across the cell cycle (S12C Fig). Collectively these data establish AUK2 as having BSF nuclear genome maintenance functions, potentially acting during replication and mitosis. The non-essentiality of AUK2 *in vitro* suggests a subservient or distinct function from AUK1, though recent data suggest AUK2 may be critical during growth in mice [99].

### Conclusions

In this work we describe the first synthetic-lethality whole genome and protein kinase-focussed RIT-seq screens applied to understand damage response pathways in *T*. *brucei*. MMS RIT-seq revealed multiple previously unexamined pathways that allow *T*. *brucei* to survive alkylation damage, with considerable overlap in the number and character of these pathways relative to *D*. *melanogaster* and yeast. Many of the MMS damage response pathways act in *T*. *brucei* genome maintenance, including repair, replication and telomere protection, but even within these well characterised pathways we reveal unexplored repair activities, including novel DNA helicases and translesion DNA polymerases. In addition, we reveal many putative MMS damage response genes that are currently annotated ‘hypothetical’, raising the possibility that *T*. *brucei* or kinetoplastid-specific survival functions are present. Finally, this study predicts ~30 PKs whose loss sensitises BSF *T*. *brucei* to MMS exposure. This number represents ~16% of the *T*. *brucei* kinome [107] and spans ~11 functional classes, suggesting widespread and unanticipated roles for PKs in responding to MMS damage. Of the PKs predicted from the screens, three are repair-associated PIKKs and one a repair-associated TLK, and we have validated eight further novel damage response PKs belonging to four classes, three of which assume greater importance to survival in mice [99]. Thus, our study uncovers a range of conserved and novel DNA repair factors, signalling factors and pathways that operate in trypanosomatids and highlights the flexibility of RNAi-based synthetic lethal screens for study of gene function in *T*. *brucei*.

## Materials and methods

### Parasite culture

BSF RNAi cell lines derived from the *T*. *brucei* strain 2T1 [108] were cultured at 37 ^o^C in 5% CO_2_ in HMI-9 medium supplemented with 10% (v/v) tetracycline-free foetal calf serum (Sigma-Aldrich) and 1% (v/v) penicillin-streptomycin solution. Cell lines were maintained in 5 μg.ml^-1^ phleomycin and 5 μg.ml^-1^ hygromycin. Cells lines expressing myc tagged proteins were grown in 10 μg.ml^-1^ of blasticidin. For all other BSF cell lines derived from WT Lister 427 cells, HMI-9 medium was supplemented with 20% (v/v) foetal calf serum. Null mutants, heterozygote cell lines and heterozygote cell lines expressing tagged proteins were maintained in the appropriate drug-free medium for no longer than 4 weeks continuous culture. Endogenous epitope tagging of the genes was performed using PCR with the oligonucleotide primer sequences detailed in S5 Table. To N-terminally 12-myc tag Tb927.11.1180, a modified pEnT6B construct [109]was used (kindly provided by R.Devlin). Cloning was performed as described in Devlin et al. [47]. The remaining PKs were C-terminally tagged using the vector pNATx12myc [92].

### Library preparation and sequencing

The whole genome RIT-seq approach was adapted from the protocol described in [31]. Pooled RNAi target fragments were amplified from genomic DNA extracted from the *T*. *brucei* populations using primers LIB2f (TAGCCCCTCGAGGGCCAGT), LIB2r (GGAATTCGATATCAAGCTTGGC) and 21 cycles at the following conditions: 95°C for 30 seconds, 57°C for 30 seconds, and 72°C for 90 seconds. The amplified PCR products ranged in size from 200 bp to 1.6 kbp, as evaluated by agarose gel electrophoresis (S1 Fig). The PCR products were cleaned up using the Qiagen QIAquick PCR purification kit then enzymatically fragmented, size selected to ~220 bp and sequencing libraries constructed, following standard protocols for Thermo Scientific Ion Proton sequence library preparation. The RNAi fragment libraries were sequenced on a Thermo Scientific Ion Torrent Proton platform using the 200 base pair sequencing kit.

For the kinome-focused RIT-seq, RNAi cell lines were generated as previously described [91]. RNAi lines were pooled, initially into 9 pools each containing 19–25 cell lines and frozen. These pools were then defrosted and further pooled to make a culture with all PK RNAi cells, which was again frozen. To perform the RIT-seq the whole kinome pool of cells was defrosted, grown for 24 h and diluted, in triplicate, to 1 x 10^5^ cells.ml^-1^ in 100 ml. Each 100 ml culture was then split into two 50 ml flasks and grown for 24 h with or without addition of tetracycline (1 μg.ml^-1^). The induced and uninduced control cultures were then again diluted to 1 x 10^5^ cells.ml^-1^ and grown for 120 h, reducing cell density to 1x10^5^ cells ml^-1^ every 24 h and sampling 1x10^7^ cells daily for genomic DNA prior to dilution. At the start of the 120 h growth three parallel cultures were derived from each of the induced and uninduced cultures: one in which no MMS was added, one in which 0.0002% MMS was added to the medium, and one with 0.0003% MMS added. To recover the RNAi target sequences from the populations, a single universal primer (5’- TAATGCCAACTTTGTACAAA-3’) was used. The primer was barcoded with 14 different 6-nucleotide tags that permitted combining equal amounts of PCR products in a single sequencing sample. Reads were assigned to each experimental condition later *in silico*. 10 ng of genomic DNA obtained per sample was PCR- amplified in a 50 μl reaction using Q5 High-Fidelity DNA polymerase (NEB, Ipswich, USA). The PCR program was: an initial 3 minutes at 98 ^o^C, followed by 28 cycles of 10 seconds at 98 ^o^C, 10 seconds at 64 ^o^C and 30 seconds at 72 ^o^C, with a final extension step at 72 ^o^C for 10 minutes. PCR products were cleaned up with a Minelute PCR purification kit (Qiagen, Venlo, Netherlands). Groups of 14 barcoded PCR products were pooled in a single sequencing sample, and 400 ng processed according to Illumina Miseq library protocols.

### Mapping

To map the RNAi reads, 'virtual' chromosomes were generated in silico by concatenating sequences of interest (e.g. the complete transcripts recorded in the TriTrypDB database for the whole genome approach or the 183 amplicons relevant to the kinome experiment), each separated by a buffer sequence of 15 random bases. The coordinates of each sequence were recorded and their artificial chromosome sequence location indexed for use in Bowtie2 (short-read alignment software). The assignment of reads to particular experimental conditions was performed by use of the Illumina bar-coding methodology in the case of the genomic experiments, and a combination of the bar-coding methodology and the presence of primer specific hexamers in the case of the kinome experiment. Single end reads (IonTorrent) or the forward sense reads (Illumina) generated from each sample containing the RNAi insert were selected by the presence of a 9 base diagnostic tag [GCCAACTTT], derived from the universal primer, allowing for 1 base mismatch (insertion, deletion or substitution). Selected reads were then mapped to the artificial chromosome with Bowtie2 (local mode alignment, default parameters). The “.sam” format files thus generated were parsed and the coordinates to which the reads mapped were recorded. Mapped reads were assigned to their appropriate PK gene using indexes generated above. A read was assigned if it lay entirely within a sequence of interest, or overlapped the ends of such a sequence. In each replicate, accumulated read abundances were normalized by multiplying raw counts 10^6^ times, dividing by the sum of total valid reads accepted for analysis in the whole sample and rounding to the next integer.

### Growth, cell cycle analysis and western blotting after RNAi induction

For growth analysis of cell lines targeting individual PK genes, cell cultures were adjusted to 1 x 10^4^ cells.ml^-1^ and the flask was split in two. To induce RNAi, tetracycline (diluted in 50% ethanol) was added (final concentration of 1 μg.ml^-1^) to one flask. Both flasks were mixed and 1.2 ml of culture aliquoted into a well in a 24 well plate, assessing cell density over 72–96 h using a Neubauer improved haemocytometer. For UV exposure, cultures were set up and RNAi induced (or not) as described above for 24 hours, after which 2 ml of each culture was aliquoted into a 6 well plate and exposed to the required UV dose (1500 J/m^2^) using a Stratalinker UV Crosslinker 2400 (Stratagene; the lid of the plate was removed during UV exposure). After UV exposure, 1.2 ml of each culture was aliquoted into a 24 well plate. To examine growth in other forms of damage, induced or uninduced cells were aliquoted into a 24 well plate as before and 0.0003% MMS (from a 0.1% stock), 0.1 μg.ml^-1^ phleomycin (from a 20 mg.ml^-1^ stock) or 0.06 mM hydroxyurea (from a 200 mM stock) added to the 1.2 ml cultures. Cell density was plotted with the error bars showing SEM of three independent experiments, except in the case of the growth curve performed for Tb927.7.960, which was performed twice. Statistical significance was assessed in Prism (GraphPad, v.5) using a Mann-Whitney U test or an unpaired t-test (for Tb927.7.960).

For cell cycle analysis, cultures were adjusted to a density of 1 x 10^5^ cells.ml^-1^ and split into two flasks, one of which was RNAi induced as described above. The flasks were further split in two and MMS (to a concentration of 0.0003%) was added to two of them (induced and uninduced). Cells were harvested by centrifugation at the indicated time points following induction, fixed in 4% Paraformaldehyde (PF) and stained with DAPI (see immunofluorescence). The ratio of N- and K-DNA was determined for over 200 cells/timepoint for three independent experiments. To evaluate levels of γH2A or myc-tagged proteins by western blotting, over 2.5 x 10^6^ cells were harvested by centrifugation at 1620 g for 10 mins at room temperature. The supernatant was removed and the pellets re-suspended in an appropriate amount of 1x protein loading buffer (PLB: 250 μl 4x NuPAGE LDS sample buffer [Invitrogen], 750 μl 1x PBS and 25 μl β-mercaptoethanol) to permit the loading of 2.5 x10^6^ cells per 10 μl and denatured at 100 ^o^C for 10 mins. Samples were stored at -20 ^o^C until required. For high molecular weight proteins, 20 μl 2x Roche Complete Mini protease inhibitor cocktail tablets was added to the loading buffer. Cell lysates were separated by SDS-PAGE using the following NuPAGE Novex pre-cast gels: 4–12% Bis-Tris, 10% Bis-Tris, 12% Bis-Tris or 3–8% Tris-acetate gels. The appropriate gel was selected based on protein size and was run as per the manufacturer’s instructions. For blotting on to PVDF membrane (Amersham Bio), proteins from the SDS-PAGE gel were transferred using a Mini Trans-Blot Cell (Bio-Rad). Transfer was performed by electrophoresis at 100 V for 2 hrs or, for high molecular weight proteins, overnight at 4 ^o^C. The membrane was incubated for 10 mins in the dark with Ponceau-S solution (Sigma) to confirm transfer of proteins had occurred. After transfer, membranes were washed once in 1x PBST (PBS, 0.01% Tween-20 [Sigma]) for 10 mins then incubated for 1 hr in blocking solution (1x PBST, 5% Milk powder [Marvel]) or, if required, overnight at 4 ^o^C. Next the membrane was rinsed for 10 mins in 1x PBST and placed in blocking buffer containing the required primary antisera for one hour (rabbit antiserum recognising phosphorylated γH2A was used at a 1:1000 dilution; mouse anti-myc antiserum (Millipore) was used at 1:7000; mouse anti-EF1a (Millipore) was used at 1:20000). The membrane was then rinsed once in 1x PBST for 20 mins and placed in blocking solution containing the appropriate secondary antisera for one hour (HRP-conjugated goat anti-mouse antiserum was used at 1:3000, and HRP-conjugated goat anti-rabbit antiserum was used at 1:5000; both ThermoFisher). After this, the membrane was washed in 1x PBST for 30 mins and SuperSignal West Pico Chemiluminescent Substrate (Thermo-Fisher) or ECL Prime Western Blotting Detection Reagent (Amersham) added and incubated in the dark for 5 mins. The membrane was then exposed to an X-ray film (Kodak) or an ECL Hyperfilm (Amersham) for ~1 sec to overnight and the film developed using a Kodak M-25-M X-omat processor. For western quantification, the following modifications were applied. Westerns were blocked in 5% milk powder in 1x PBS overnight at 4 ^o^C. Chameleon Duo Pre-Stained Protein Ladder (2 μl; Li-Cor) was loaded to confirm protein sizes. The following secondary antibodies were used: IRDye 680 goat anti-mouse and IRDye 800 goat anti-rabbit (both 1:10000, Li-Cor). Before imaging after the final 1x PBST wash, the membranes were subject to a final wash in 1x PBS. The images were captured using an Odyssey CLx Imager (Li-Cor) using the in-built software (ImageStudio) to obtain the intensities of each band. The fold change was calculated by normalising each sample to the loading control and calculating the relative fold change to the control sample. The numerical data were analysed using GraphPad Prism 5.0.

### Generation of null mutants

Heterozygous (+/-) and homozygous (-/-) mutants of *auk2* were generated by replacing most of the gene’s ORF with a selective drug marker. Two modified versions of the pmtl23 plasmid (gift, Marshall Stark, University of Glasgow), containing either the blasticidin or neomycin resistance genes, were used. Details of the cloning approach are described in [47]. To generate the knockout constructs, PCR was performed from *T*. *brucei* genomic DNA to amplify the 5’ or 3’ ORF flanks using primers 141 and 142, and 143 and 144, respectively (S6 Table). RNA in the mutants was analysed by RT-PCR, amplifying a region of the ORF with primers 147 and 148, or by qRT-PCR with primers OL31 and OL32. RNA was extracted from cells using the Qiagen RNeasy kit, and cDNA synthesis was performed using random primers and the Primer Design Precision nanoScript Reverse Transcription kit (Primer Design), according to manufacturer’s instructions. For qRT-PCR, each analysis was performed as a technical triplicate. Master mix was as follows (prepared at 4 ^o^C, but not in direct contact with ice): 12.5 μl SYBR Green PCR Master Mix (Applied Biosystems), 5 μl RNase free ddH_2_0 (Qiagen), 2.5 μl of each primer (300 nM stock) and 2.5 μl of the appropriate cDNA. The master mix was pipetted into a MicroAmp Optical 96-well reaction plate (Thermo Fisher). Actin (primers OL29 and OL30) were used as an endogenous control, and ddH_2_0 (RNase free) was used as a negative control. AB 7500 RT PCR system thermocycler was used and conditions for all reactions were 50 ^o^C for 2 min, 95 ^o^C for 10 min, and 40 cycles of 95 ^o^C for 15 sec followed by 60 ^o^C for 1 min, with a final dissociation step of 95 ^o^C for 15 secs, 60 ^o^C for 1 min, 95 ^o^C for 15 secs and, finally, 60 ^o^C for 15 secs. The data was processed as detailed in the Applied Biosystems manual using the ddCt approach.

### Immunofluorescence

For immunofluorescence and DAPI analysis, approximately 2x 10^6^ cells were harvested by centrifugation (405 g for 10 mins). The pellet was washed in 1x PBS by centrifugation (405 g for 3 mins), the supernatant removed and the pellet re-suspended in ~50 μl 1xPBS. The cells were settled for 5 mins on a 12 well glass (Menzel-Gläser) slide treated with Poly-L-Lysine (Sigma). A wax barrier was drawn around the wells using a PAP pen (Life Technologies). The supernatant was removed and 25 μl 4% formaldehyde (FA) was added for 4 mins. The FA was then removed and the cells washed 3 times in 50 μl 1x PBS for 5 mins. To stain DNA, 5 μl of DAPI (Southern Biotech) was added to each well and incubated at room temperature for 4 mins. A coverslip was then added and sealed with nail varnish. Slides were stored in the dark at 4 ^o^C. For immunofluorescence cells were permeabilised with 25 μl 1x PBS/Triton X-100 (Thermo Scientific) for 10 mins. To neutralise free -aldehyde groups, 100 mM glycine in PBS was added for 20 mins. The wells were then washed three times in 1x PBS for 5 mins. The wells were blocked for 1 hr with 25 μl blocking solution (1% BSA [Sigma], 0.2% Tween-20 in 1 x PBS) in a wet chamber. Afterwards, 25 μl of the required primary antiserum diluted in blocking solution was then added and incubated for 1 hr in a wet chamber: rabbit anti-RAD51 at 1:1000; rabbit anti-γH2A at 1:1000; and AlexaFluor 488 conjugated mouse-anti-myc (Millipore) at 1:500. The wells were then washed 2 x with 1 x PBS for 5 mins. 25 μl of the appropriate secondary antisera (always goat AlexaFluor 488 or 594 anti-mouse or anti-rabbit from Millipore at 1:1000) were added to each well and then incubated for 1 hr in a wet chamber, after which the cells were washed three times with 1x PBS for 5 mins. For immunofluorescence requiring anti-KMX-1 antiserum, blocking was performed for 1 hr in 25 μl PBS. The cells were then DAPI stained and the slides stored as described above. Standard images were captured on an Axioskop 2 (Zeiss) fluorescence microscope, using a 63 x DIC magnification lens and ZEN software package (Zeiss). Alternatively, images were captured on an Olympus IX71 DeltaVision Core System (Applied Precision, GW) using a 1.40/100 x lens and acquired using the SoftWoRx suite 2.0 software (Applied Precision, GE). Z-stacks were acquired of varying thickness (no more than 10 μm); images were de-convolved (conservative ratio; 1024x1024 resolution) by the SoftWoRx software. Super-resolution structure illuminated images were captured on an Elyra PS.1 super resolution microscope (Zeiss). Raw images were acquired using the provided ZEN Black Edition Imaging Software tool (Zeiss). The images were then aligned to the channel alignment files generated on the day of imaging using the same software. All images were processed in ImageJ/Fiji (http://fiji.sc/Fiji). For most images, excluding the ones used for quantification of the DAPI signal, both the contrast and brightness of the DAPI signal was enhanced to improve visualisation. For all images, the background was subtracted and suitable false colours were assigned to the fluorescence channels.

### Transmission electron microscopy

Approximately 5 x10^6^ cells were fixed in 2.5% glutaraldehyde and 4% PF in 0.1 M sodium cacodylate buffer (pH 7.2) then post-fixed for 45 mins in 1% osmium tetroxide and 2.5% potassium ferrocyanide (pH 7.3) in 0.1 M sodium cacodylate buffer in the dark. The cells were washed several times with 0.1 M cacodylate buffer and the samples stained (en bloc) with 2% aqueous uranyl acetate the dehydrated in acetone solutions (30, 50, 70, 90 and 100%). The samples were then embedded in Epon resin and sectioned (ultrathin sectioning). The samples were visualised on a Tecnai T20 transmission electron microscope (FEI, Netherlands).

### Data access

Sequences used in the mapping have been deposited in the European Nucleotide Archive (accession numbers PRJEB19516 and PRJEB19634; http://www.ebi.ac.uk/ena). RITseq data will be hosted at TriTryDB (http://tritrypdb.org/tritrypdb/) in an upcoming release.

## Supporting information

S1 Table Average Ion Torrent reads that map to each *T*. *brucei* gene (tritrypDB gene ID and annotated product description provided) in RNAi target PCR libraries prepared from *T*. *brucei* cells grown in tetracycline (Tet) for 1 day (control, C), and after 5 days in Tet without (T) or with (Tm) addition of methyl methanesulphonate (MMS) for the final 4 days growth. Reads are normalised to CDS length and total number of reads (NGP denotes no mapped reads were detected), and ratios of the reads in the different samples, as well as a summary of the effects predicted by the ratios, are provided. Data are ordered from low to high Tm/T ratios.
(XLSX)

S2 Table Tm/T read ratios (see S1 Fig) for *T*. *brucei* genes are shown in 10 worksheets, which correspond to a number of named functional classes; tritrypDB gene ID and annotated product descriptions are provided, as well as functional commentary. (XLSX)

S3 Table All genes with a Tm/T ratio (see S1 Fig) of less than 0.5 are shown in worksheet 1, including tritrypDB gene ID and annotated product descriptions, plus a functional commentary. Worksheet 2 shows Gene Ontology (GO) categories, in two functional classifications, which display significant enrichment in number of genes in the <0.5 gene set relative to the expected number of genes based on total gene number in the genome. Worksheet 3 shows networks of genes that may act in the response to MMS damage, comparing RNAi data in the presence of MMS in *D*. *melanogaster* [29] and in *T*. *brucei* bloodstream form cells (this study).
(XLSX)

S4 Table MMS RIT-seq analysis of the *T*. *brucei* kinome. Worksheet 1 shows all protein kinase (PK) genes examined (tritrypDB IDs provided), detailing the ratio of reads in Tet-induced cells treated with either 0.0002% or 0.0003% MMS relative to Tet-induced cells not exposed to MMS cells (TM2/TM0 and TM3/TMO, respectively); ratios are shown after 2, 3, 4 or 5 days growth (parentheses). For comparison, read depth ratios are also shown in Tet-induced, MMS treated cells relative to MMS treated cells without Tet induction (TM2/CM2 and TM3/CM3, respectively). Cells are coloured as follows: black = ratios below detection; blue = ratios between the minimum detectable level and 0.3; green = ratios between 0.3–0.6; white = ratios of 0.6 or above; red = ratios above 1.0. The final columns provide a colour coding summary of any read changes in days 1, 2, 3, 4 and 5 relative to day 0 (pre- RNAi induction) in the absence of Tet or MMS addition (tp 1–5, control progression), and in Tet-induced cells without MMS at days 2, 3, 4 and 5 relative to day 1 post-induction (tps 2–5, -MMS); colours correspond to the same fold changes as above. Worksheet 2 describes PK genes predicted to display elevated loss of reads after RNAi in the presence of MMS relative to its absence. Worksheet 3 compares the kinome-focused MMS RITseq ratios with those seen in the whole genome MMS RITseq, detailing the PK class, name (if available) and a commentary on experimental validation of predicted damage-associated PKs.
(XLSX)

S5 Table Primers used to add epitope tags to protein kinase genes; predicted protein name and tritrypDB gene ID are provided. (XLSX)

S6 Table Primers used in the generation and analysis of *AUK2* null mutants. (XLSX)

S1 Fig PCR amplification of RNAi target fragments. RNAi target fragments were PCR-amplified from two replicates of bloodstream form *T*. *bruce*i cells in which RNAi had been induced with tetracycline (Tet+) for 5 days with (MMS+) or without (MMS-) growth in 0.0003% methyl methanesulphonate for the final 4 days. Products were analysed by electrophoretic separation on a 1.5% w/v agarose gel and sizes are compared with a ladder.
(PDF)

S2 Fig Comparison of the total sequence reads across two independent RITseq screens. The total number of sequence reads which mapped to a minimal *T*. *brucei* genome were compared at day six [19] and day five (this study) post-RNAi induction. The coefficient of determination is shown.
(PDF)

S3 Fig MMS RIT-seq prediction of helicases in *T*. *brucei*. Scatter plots showing the ratio of mapped RNAi target-specific reads for every gene (grey dots) in the RNAi-induced, MMS-treated population relative to the RNAi-induced, untreated population (MMS+/MMS-); gene location within the 11 megabase chromosomes is shown. All predicted *T*. *brucei* helicase genes are separated into putative RNA (blue) and DNA helicases (red), or those whose substrate is unclear (black). Arrows highlight DNA helicases discussed in the text, and PIF1 family helicases are highlighted in orange.
(PDF)

S4 Fig Preliminary analysis of four whole-genome MMS RITseq-predicted damage associated protein kinases. **A.** Individual tetracycline (Tet) inducible RNAi cell lines were generated for two PK genes (identified by gene ID and name) and their growth assessed by counting parasite density every 24 hrs for 96 hrs. Growth was assessed in the absence (-) and presence (+) of MMS (0.0003% v/v) and with (+) or without (-) Tet RNAi induction. Mapping profiles of the same genes are shown alongside, after RNAi and growth with (+) or without (-) 0.0003% MMS. **B.** Mapping profiles of two further PK genes (IDs provided).
(PDF)

S5 Fig *in vitro* cell cycle analysis of the effects of RNAi against genome-wide PK damage-associated candidates. Cells were collected from tetracycline induced (1 μg.ml^-1^; +) or uninduced (-) cultures at 24 and 48 hrs. Cells were also harvested from cultures induced or uninduced in the presence of 0.0003% MMS (v/v). Cells were fixed and stained with DAPI. The number of 1N1K, 1N2K, 2N2K and ‘other’ (including 0N1K, zoid) cells were counted and expressed as a percentage of the total population. The error bars represent ±SEM (n = 3, > 200 cells counted per experiment). Significance was calculated by comparing ‘other’ cells from the non-induced individually with ‘other’ cells from the induced cells using a Mann Whitney U test (one tailed). (*) = p<0.05.
(PDF)

S6 Fig Preliminary analysis of three kinome MMS RITseq-predicted damage associated protein kinases. Individual tetracycline (Tet) inducible RNAi cell lines were generated for the PK genes (identified by gene ID and name, if available) and their growth assessed by counting parasite density every 24 hrs for 96 hrs. Growth was assessed in the absence (-) and presence (+) of MMS (0.0003% v/v) and with (+) or without (-) Tet RNAi induction.
(PDF)

S7 Fig *in vitro* cell cycle analysis of the effects of RNAi against kinome MMS RITseq-predicted PK damage-associated candidates. Cells were collected from tetracycline induced (1 μg.ml^-1^; +) or uninduced (-) cultures at 24 and 48 hrs. Cells were also harvested from cultures induced or uninduced in the presence of 0.0003% MMS (v/v). Cells were fixed and stained with DAPI. The number of 1N1K, 1N2K, 2N2K and ‘other’ (including 0N1K, zoid) cells were counted and expressed as a percentage of the total population. The error bars represent ±SEM (n = 3, > 200 cells counted per experiment). Significance was calculated by comparing ‘other’ cells from the non-induced individually with ‘other’ cells from the induced cells using a Mann Whitney U test (one tailed). (*) = p<0.05. Gene IDs and names are provided for the PKs genes analysed.
(PDF)

S8 Fig Generation and analysis of *T*. *brucei* bloodstream form *AUK2* null mutants. **A.** Schematic of the strategy for *AUK2* gene replacement by homologous recombination of a construct containing cassettes encoding resistance to blasticidin (blasticidin S deaminase; BSD) or G418 (neomycin phosphotransferase; NEO). Crosses indicate recombination on the gene’s untranslated regions (UTRs); arrows show primers used to test drug resistant transformants by RT-PCR; and Tub and Actin denote sequences from the tubulin and actin loci used to direct mRNA processing of the BSD and NEO ORFs after integration. **B.** An agarose gel of end-point RT-PCR (using primers in A) performed on cDNA (+) from wild type (WT 427), AUK2 heterozygous mutants (+/-) and two homozygous mutants (-/- CL1 and CL2). A control reaction on RNA not treated with reverse transcriptase (-) is included, as is PCR on genomic DNA or distilled water. All samples were also subjected to (RT)PCR with primers recognising a control ORF (Tb927.9.6560) and generating a similar sized PCR product. **C.** ΔΔCt RT-qPCR, using the same cDNA in B and primers AUK2RTFW2 and AUK2RTRV1, to evaluate AUK2 RNA levels in WT, +/- and -/- cells. Expression of AUK2 in the WT was arbitrarily set to 100% and levels in the other samples are expressed relative to that. **D.** Cell cycle analysis of WT, +/- and -/- cells after DAPI for visualisation of the kinetoplast (k) and the nucleus (n). >200 cells were counted from three independent replicates of each cell type, and the n-k configuration of individual cells expressed as a percentage of the total population. Cells that did not show any of the expected N-K configurations (1N1K, 1N2K or 2N2K) were categorised as ‘other’. Error bars represent SEM. * P<0.05 (Mann Whitney U test; comparison between WT other cells and +/- or -/- other cells). **E.** Representative images of cells from the AUK2 -/- CL1 mutant. Cells were fixed and stained with DAPI (cyan) and anti-KMX1 antiserum (to detect tubulin; magenta). Examples of the range of ‘other’ cells observed are shown above; below are cells conforming to the normal 1N1K, 1N2K or 2N2K categories. Scale bar = 5 μm.
(PDF)

S9 Fig Loss of AUK2 sensitises *T*. *brucei* to DNA damaging agents. Growth curves of either (**A**) *AUK2* heterozygous mutants (+/-) or (**B**) *auk2* -/- null mutant clone 2 (CL2) are shown compared with wildtype (WT427) cells. In all graphs cell density was monitored every 24 hrs for 72 hrs in the presence (+) and absence of MMS (0.0003%), phleomycin (PHL; 0.1 μg.ml^-1^), hydroxyurea (HU; 0.6 mM; C) or after exposure to UV (1500 J/m^2^). All graphs show mean density from three experiments; error bars denote SEM. Significant differences are shown by * (P<0.05; Mann Whitney U test). For the -/- CL2 mutants, no cells could be detected after 24 hrs growth in MMS.
(PDF)

S10 Fig Loss of AUK2 results in increased expression of γH2A. **A.** To gels, representing independent experiments, are shown of western blot analysis of γH2A in two *auk2* -/- mutants clones (CL1 and CL2) relative to an *AUK*+/- heterozygous mutant and wild type cells (WT427). Whole cell lysates were probed with anti-γH2A (below) and anti-EF1α (above; loading control) antisera. **B.** Immunofluorescence (IF) of γH2A. Cells were harvested, fixed and RAD51 localised with anti- γH2A antiserum. Representative IF images of *auk2*-/- mutants and WT cells are shown in which DAPI stained DNA is in blue and γH2A in red (cell morphology is shown by differential contrast imaging); the scale bar = 10 μm.
(PDF)

S11 Fig Loss of AUK2 is associated with increased levels of nuclear defects in bloodstream form *T*. *brucei*. **A.** >250 cells were imaged after DAPI staining (cyan) and examined for the presence of nuclear ‘blebs’, scoring wild type, *AUK2*+/-, or *auk2*-/- (two clones, CL1 and CL2) cells either as having a discernible ‘bleb’ or as having ‘no bleb’. Each category is expressed as a percentage of the total cell count. Below are representative examples of the two categories; scale bar = 5 μm, and the arrow indicates a bleb. **B.** Analysis of the mitotic spindle of 1N2K cells in WT, *AUK2*+/- and *auk2*-/- cells. Images of 1N2K cells categorised depending upon the presence (spindle) or absence of (no spindle) of a mitotic spindle, which was visualised by staining with anti-KMX-1 antiserum (green) and co-localisation with DAPI (magenta). Counts are represented as a percentage of the total cells examined; the number of cells counted is indicated above the corresponding bars. Representative images of a WT cell with an intact mitotic spindle (indicated by the white arrow), and an *auk2-*/- cell without a detectable spindle are shown; scale bar = 5 μm.
(PDF)

S12 Fig Localisation of myc-tagged AUK2 in bloodstream form *T*. *brucei*. **A.** *in vitro* growth analysis of *AUK2*+/-12myc cell lines, in which one allele of *AUK2* is C-terminally fused to 12 copies of the myc epitope and the other allele is disrupted. Growth was evaluated by measuring cell density over 96 hrs and compared with wild type (WT), *AUK2*+/- and *AUK2*+/+12myc (one *AUK2* allele myc-tagged, the other intact) cells. **B.** Quantification, after immunolocalisation of AUK2-12myc with anti-myc antiserum, of the number of *AUK2*+/-12myc (two clones, CL1 and CL2) cells with or without a discernible nuclear signal; the total number of cells counted is shown, and the two categories are represented as a percentage of the total. **C.** Intensity of nuclear DNA DAPI signal (left, blue) or anti-myc signal in immunoflouresence (right, green), was measured using ImageJ and is shown for cells with 1N1k, 1N2K and 2N2K nuclear (n) and kinetoplast (k) configurations. To perform this analysis, a region of interest (21x21 pixels) was drawn around each nucleus and the mean pixel intensity recorded (represented by a ‘dot’ on the graph). The error bars represent the median value and the interquartile range. Significance was assessed using the Kruskal-Wallis non parametric test. (*) p<0.05, (**) p<0.005 and (***) p<0.0005. n = 253 cells, n = 1. **D.** Super resolution images of AUK2-12myc localisation with anti-myc antiserum in 2N2K AUK2+/-12myc cells, demonstrating the lack of detectable nuclear signal. The graphs show fluorescence intensity (arbitrary units; AU) plotted for both the DAPI (blue) and anti-myc (green) signal. The white box represents the area from which the fluorescence intensity was measured (using ImageJ) to generate the graph. Fluorescence intensity (as AU) is plotted (X-axis) over distance (μm; Y-axis). Scale bar = 5 μm. **E.** A representative super resolution image of AUK2-12myc localisation with anti-myc antiserum after growth of AUK2+/-12myc cells for 18 hrs in 0.0003% MMS.
(PDF)
