# Supplementary figures and images for: Genome-wide and protein kinase-focused RNAi screens reveal conserved and novel damage response pathways in Trypanosoma brucei
Source: PLoS Pathog. 2017 Jul 24;13(7):e1006477. doi: 10.1371/journal.ppat.1006477 (PMC5542689; doi:10.1371/journal.ppat.1006477)

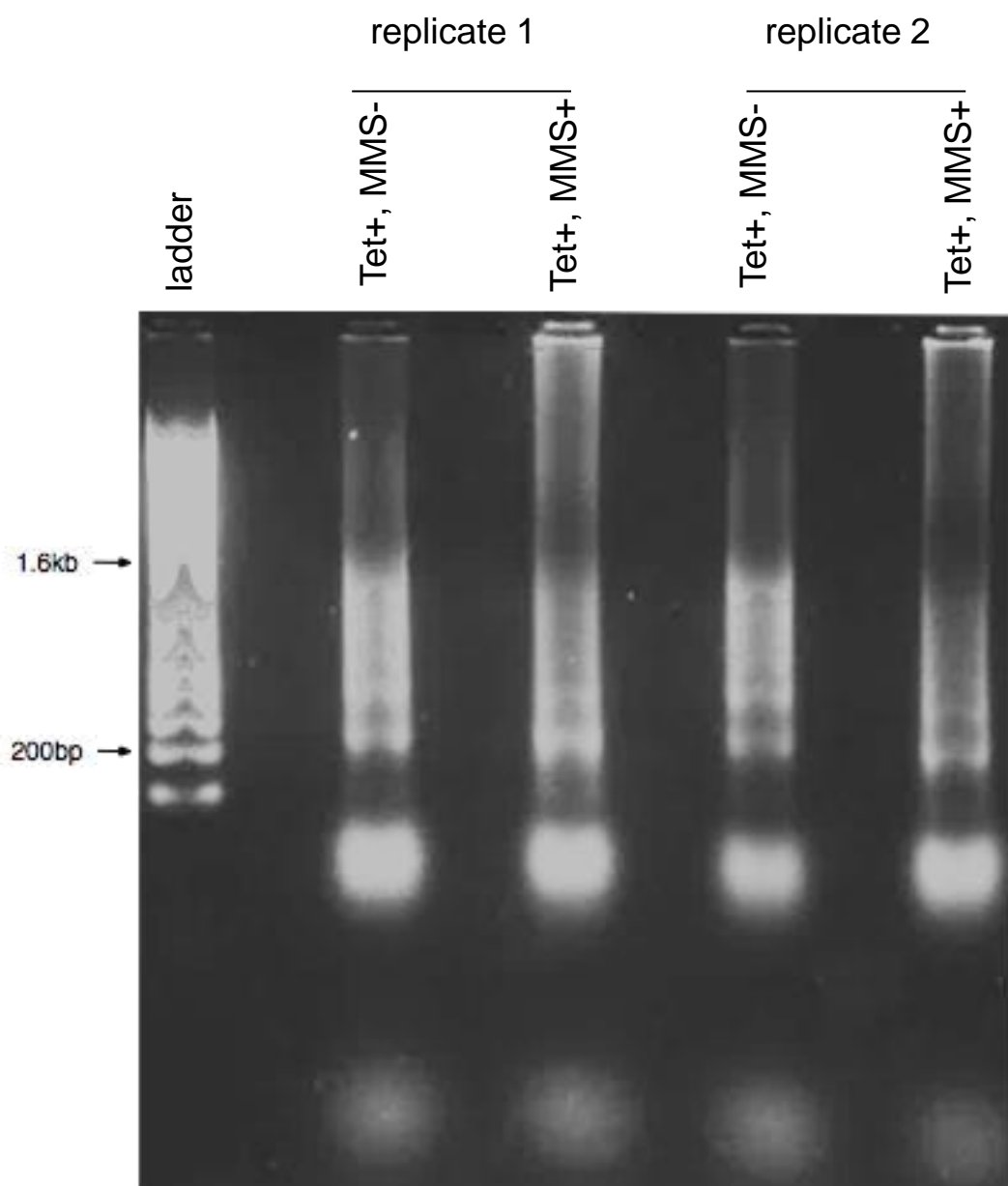

Fig.S1

Supplement: S1 Fig — RNAi target fragments were PCR-amplified from two replicates of bloodstream form T. brucei cells in which RNAi had been induced with tetracycline (Tet+) for 5 days with (MMS+) or without (MMS-) growth in 0.0003% methyl methanesulphonate for the final 4 days. Products were analysed by electrophoretic separation on a 1.5% w/v agarose gel and sizes are compared with a ladder. (PDF) [file ppat.1006477.s007.pdf]

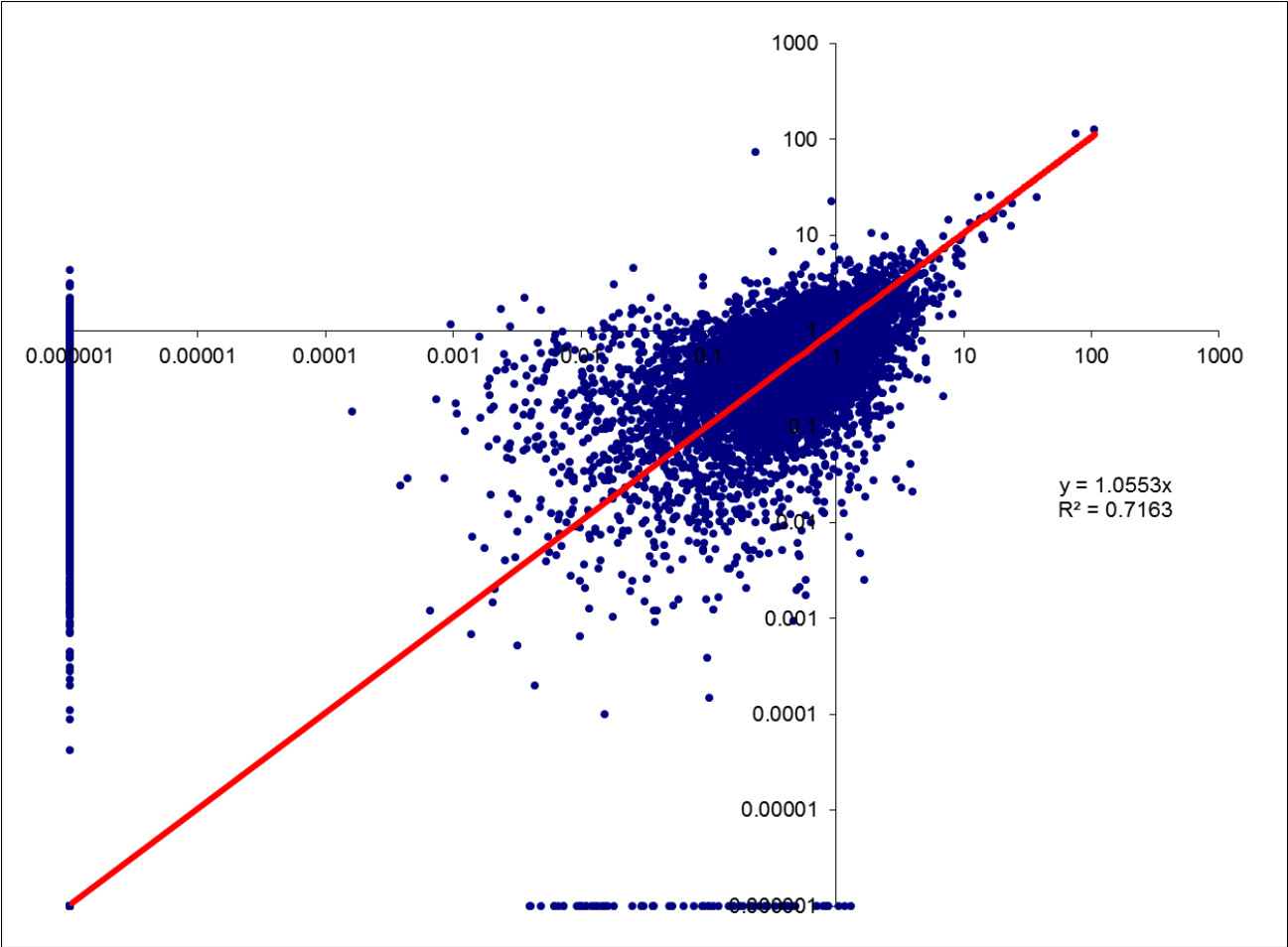

Fig.S2

Supplement: S2 Fig — The total number of sequence reads which mapped to a minimal T. brucei genome were compared at day six [19] and day five (this study) post-RNAi induction. The coefficient of determination is shown. (PDF) [file ppat.1006477.s008.pdf]

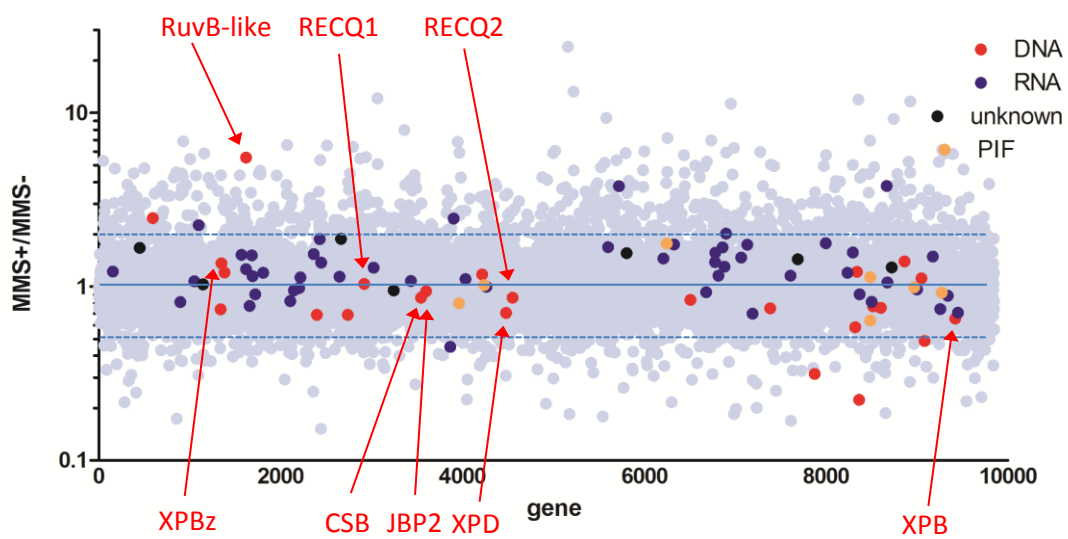

Fig.S3

Supplement: S3 Fig — Scatter plots showing the ratio of mapped RNAi target-specific reads for every gene (grey dots) in the RNAi-induced, MMS-treated population relative to the RNAi-induced, untreated population (MMS+/MMS-); gene location within the 11 megabase chromosomes is shown. All predicted T. brucei helicase genes are separated into putative RNA (blue) and DNA helicases (red), or those whose substrate is unclear (black). Arrows highlight DNA helicases discussed in the text, and PIF1 family helicases are highlighted in orange. (PDF) [file ppat.1006477.s009.pdf]

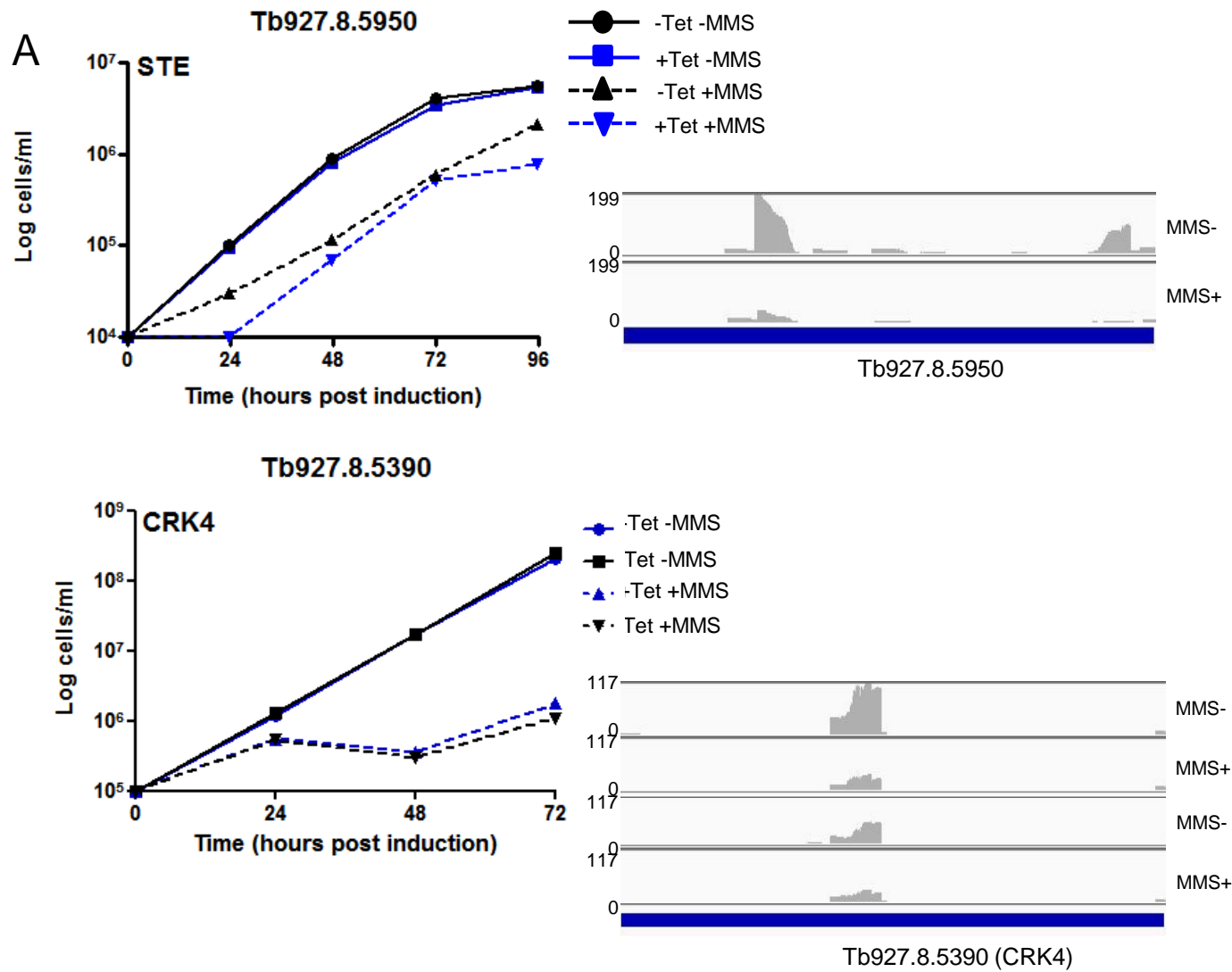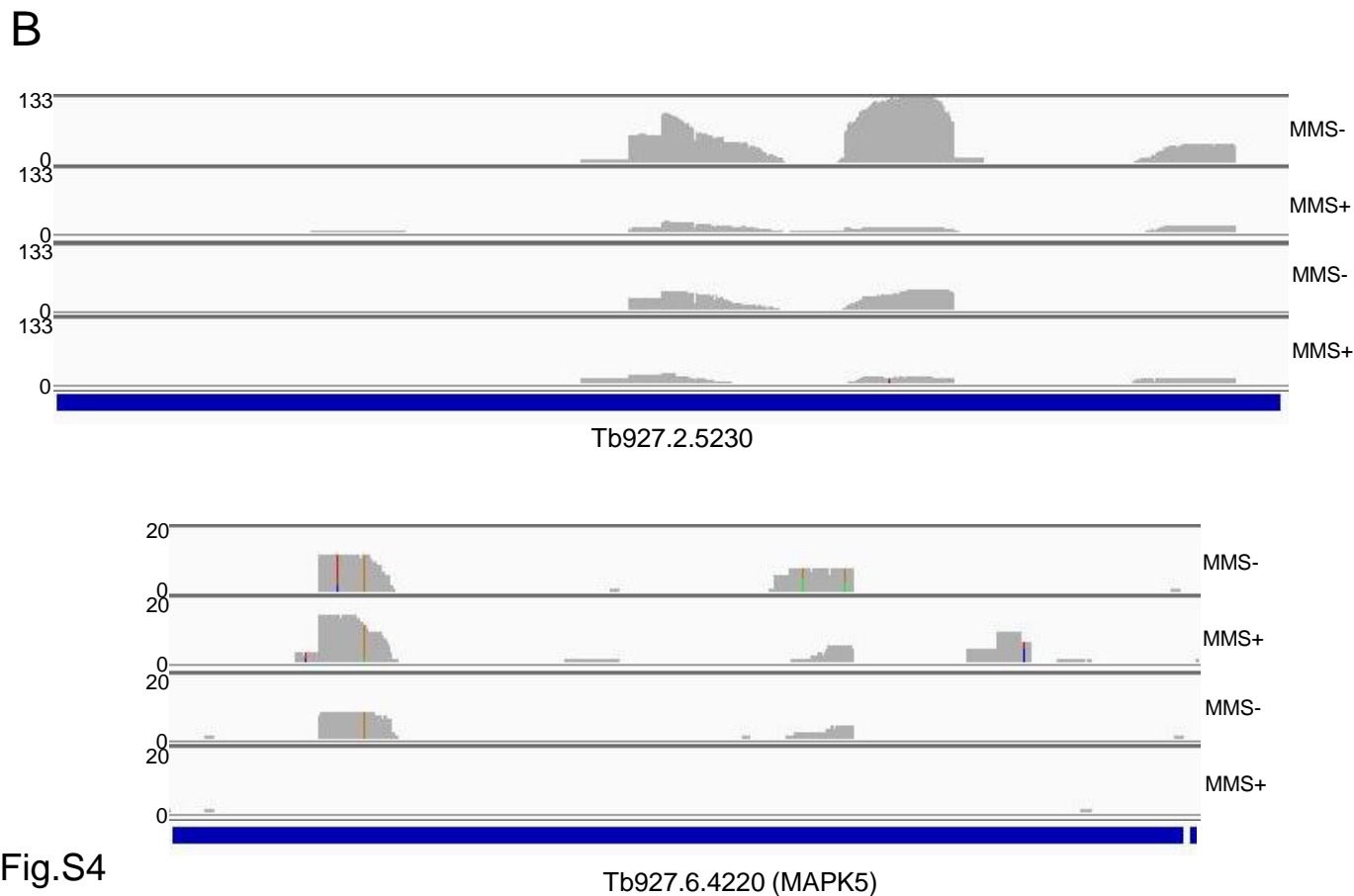

Fig.S4

Supplement: S4 Fig — A. Individual tetracycline (Tet) inducible RNAi cell lines were generated for two PK genes (identified by gene ID and name) and their growth assessed by counting parasite density every 24 hrs for 96 hrs. Growth was assessed in the absence (-) and presence (+) of MMS (0.0003% v/v) and with (+) or without (-) Tet RNAi induction. Mapping profiles of the same genes are shown alongside, after RNAi and growth with (+) or without (-) 0.0003% MMS. B. Mapping profiles of two further PK genes (IDs provided). (PDF) [file ppat.1006477.s010.pdf]

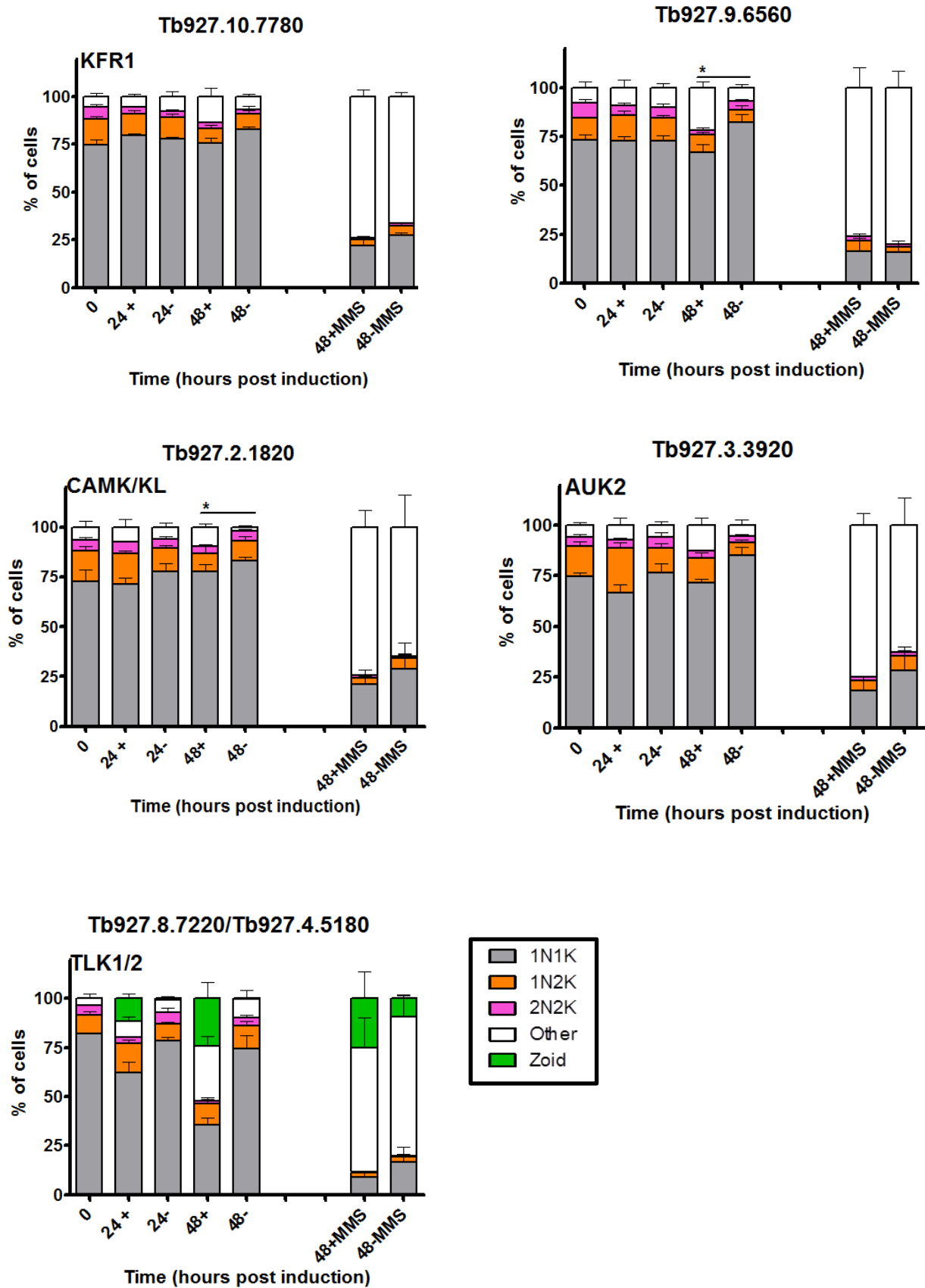

Fig.S5

Supplement: S5 Fig — Cells were collected from tetracycline induced (1 μg.ml-1; +) or uninduced (-) cultures at 24 and 48 hrs. Cells were also harvested from cultures induced or uninduced in the presence of 0.0003% MMS (v/v). Cells were fixed and stained with DAPI. The number of 1N1K, 1N2K, 2N2K and ‘other’ (including 0N1K, zoid) cells were counted and expressed as a percentage of the total population. The error bars represent ±SEM (n = 3, > 200 cells counted per experiment). Significance was calculated by comparing ‘other’ cells from the non-induced individually with ‘other’ cells from the induced cells using a Mann Whitney U test (one tailed). (*) = p<0.05. (PDF) [file ppat.1006477.s011.pdf]

A

Tb927.11.7010

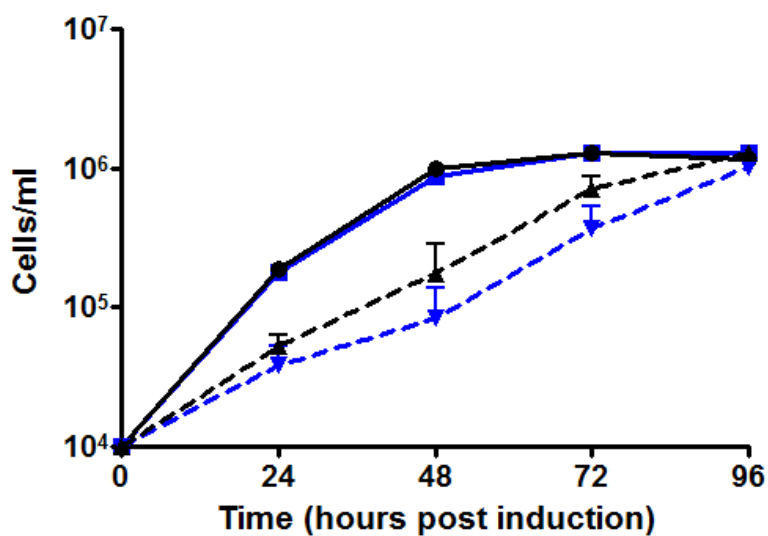

B

Tb927.6.1780

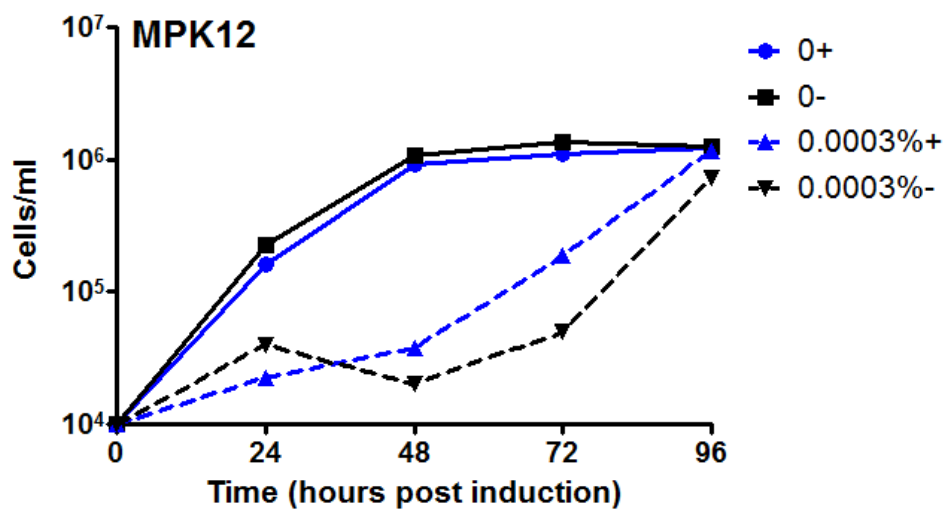

Tb927.4.5390/Tb927.8.6930

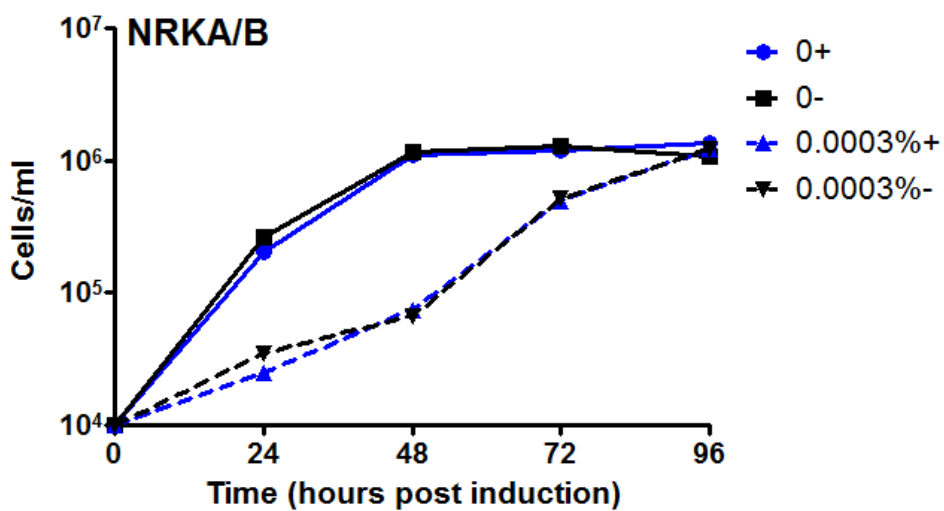

Fig.S6

Supplement: S6 Fig — Individual tetracycline (Tet) inducible RNAi cell lines were generated for the PK genes (identified by gene ID and name, if available) and their growth assessed by counting parasite density every 24 hrs for 96 hrs. Growth was assessed in the absence (-) and presence (+) of MMS (0.0003% v/v) and with (+) or without (-) Tet RNAi induction. (PDF) [file ppat.1006477.s012.pdf]

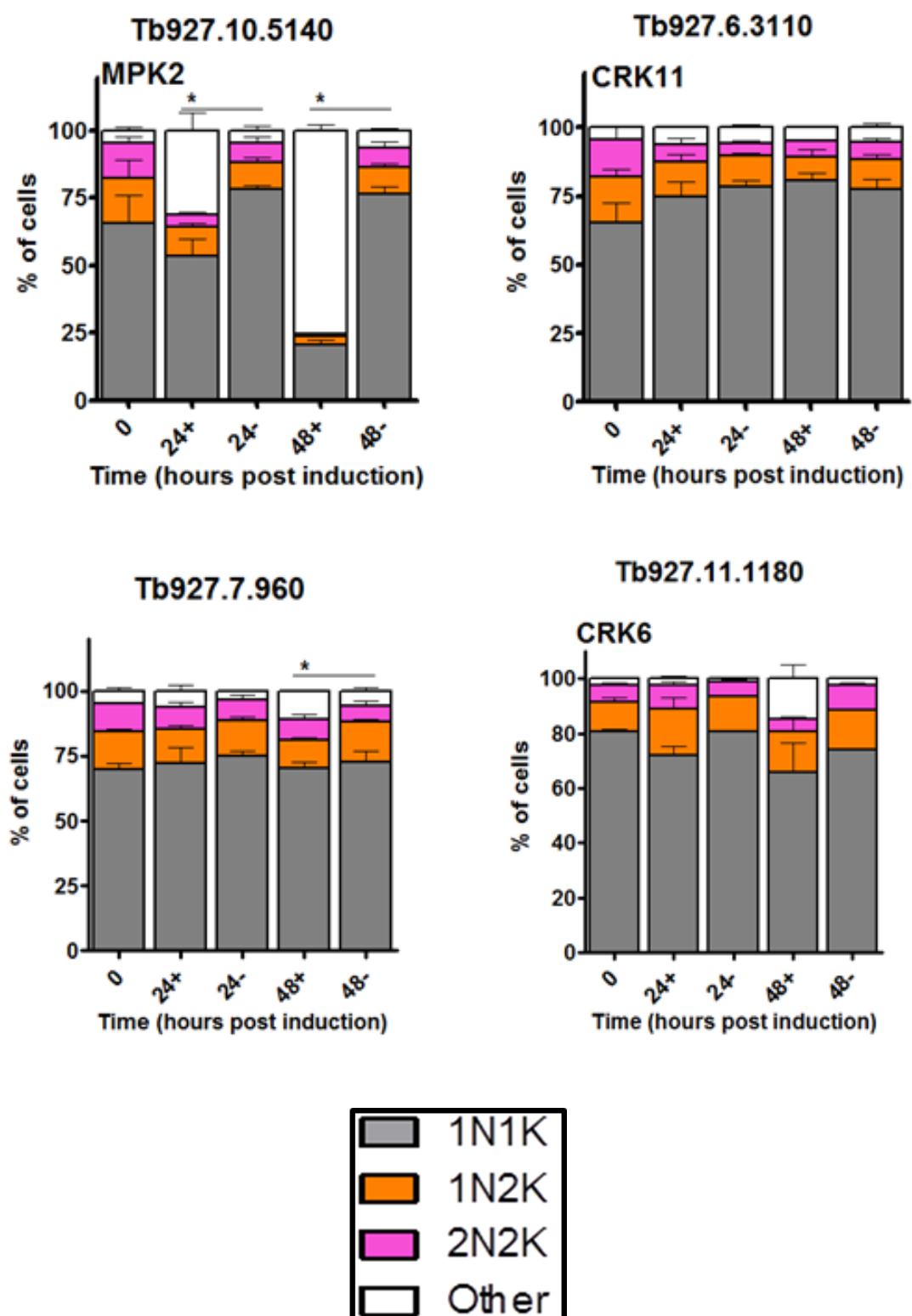

Fig.S7

Supplement: S7 Fig — Cells were collected from tetracycline induced (1 μg.ml-1; +) or uninduced (-) cultures at 24 and 48 hrs. Cells were also harvested from cultures induced or uninduced in the presence of 0.0003% MMS (v/v). Cells were fixed and stained with DAPI. The number of 1N1K, 1N2K, 2N2K and ‘other’ (including 0N1K, zoid) cells were counted and expressed as a percentage of the total population. The error bars represent ±SEM (n = 3, > 200 cells counted per experiment). Significance was calculated by comparing ‘other’ cells from the non-induced individually with ‘other’ cells from the induced cells using a Mann Whitney U test (one tailed). (*) = p<0.05. Gene IDs and names are provided for the PKs genes analysed. (PDF) [file ppat.1006477.s013.pdf]

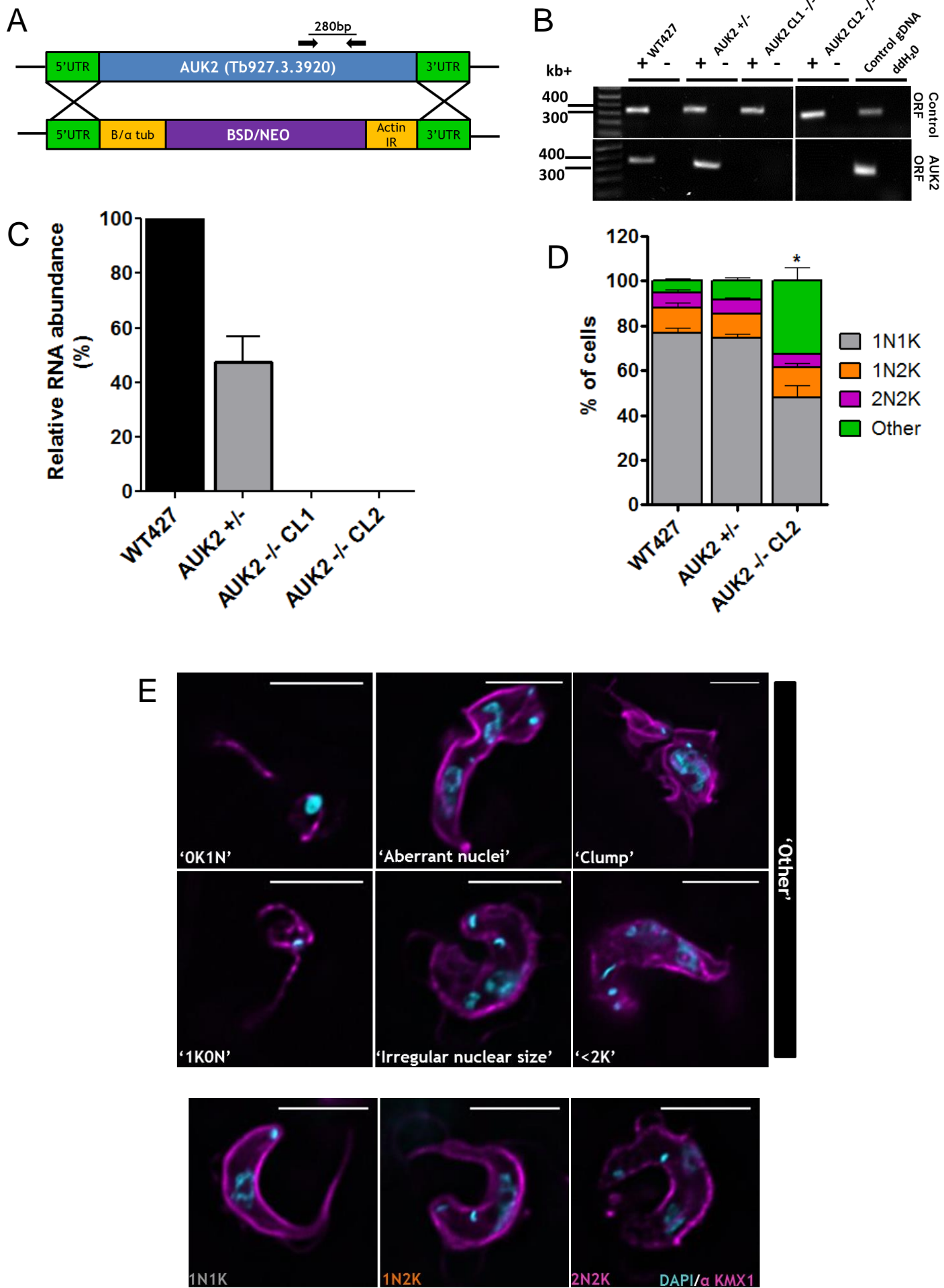

Fig.S8

Supplement: S8 Fig — A. Schematic of the strategy for AUK2 gene replacement by homologous recombination of a construct containing cassettes encoding resistance to blasticidin (blasticidin S deaminase; BSD) or G418 (neomycin phosphotransferase; NEO). Crosses indicate recombination on the gene’s untranslated regions (UTRs); arrows show primers used to test drug resistant transformants by RT-PCR; and Tub and Actin denote sequences from the tubulin and actin loci used to direct mRNA processing of the BSD and NEO ORFs after integration. B. An agarose gel of end-point RT-PCR (using primers in A) performed on cDNA (+) from wild type (WT 427), AUK2 heterozygous mutants (+/-) and two homozygous mutants (-/- CL1 and CL2). A control reaction on RNA not treated with reverse transcriptase (-) is included, as is PCR on genomic DNA or distilled water. All samples were also subjected to (RT)PCR with primers recognising a control ORF (Tb927.9.6560) and generating a similar sized PCR product. C. ΔΔCt RT-qPCR, using the same cDNA in B and primers AUK2RTFW2 and AUK2RTRV1, to evaluate AUK2 RNA levels in WT, +/- and -/- cells. Expression of AUK2 in the WT was arbitrarily set to 100% and levels in the other samples are expressed relative to that. D. Cell cycle analysis of WT, +/- and -/- cells after DAPI for visualisation of the kinetoplast (k) and the nucleus (n). >200 cells were counted from three independent replicates of each cell type, and the n-k configuration of individual cells expressed as a percentage of the total population. Cells that did not show any of the expected N-K configurations (1N1K, 1N2K or 2N2K) were categorised as ‘other’. Error bars represent SEM. * P<0.05 (Mann Whitney U test; comparison between WT other cells and +/- or -/- other cells). E. Representative images of cells from the AUK2 -/- CL1 mutant. Cells were fixed and stained with DAPI (cyan) and anti-KMX1 antiserum (to detect tubulin; magenta). Examples of the range of ‘other’ cells observed are shown above; below are cells conf [file ppat.1006477.s014.pdf]

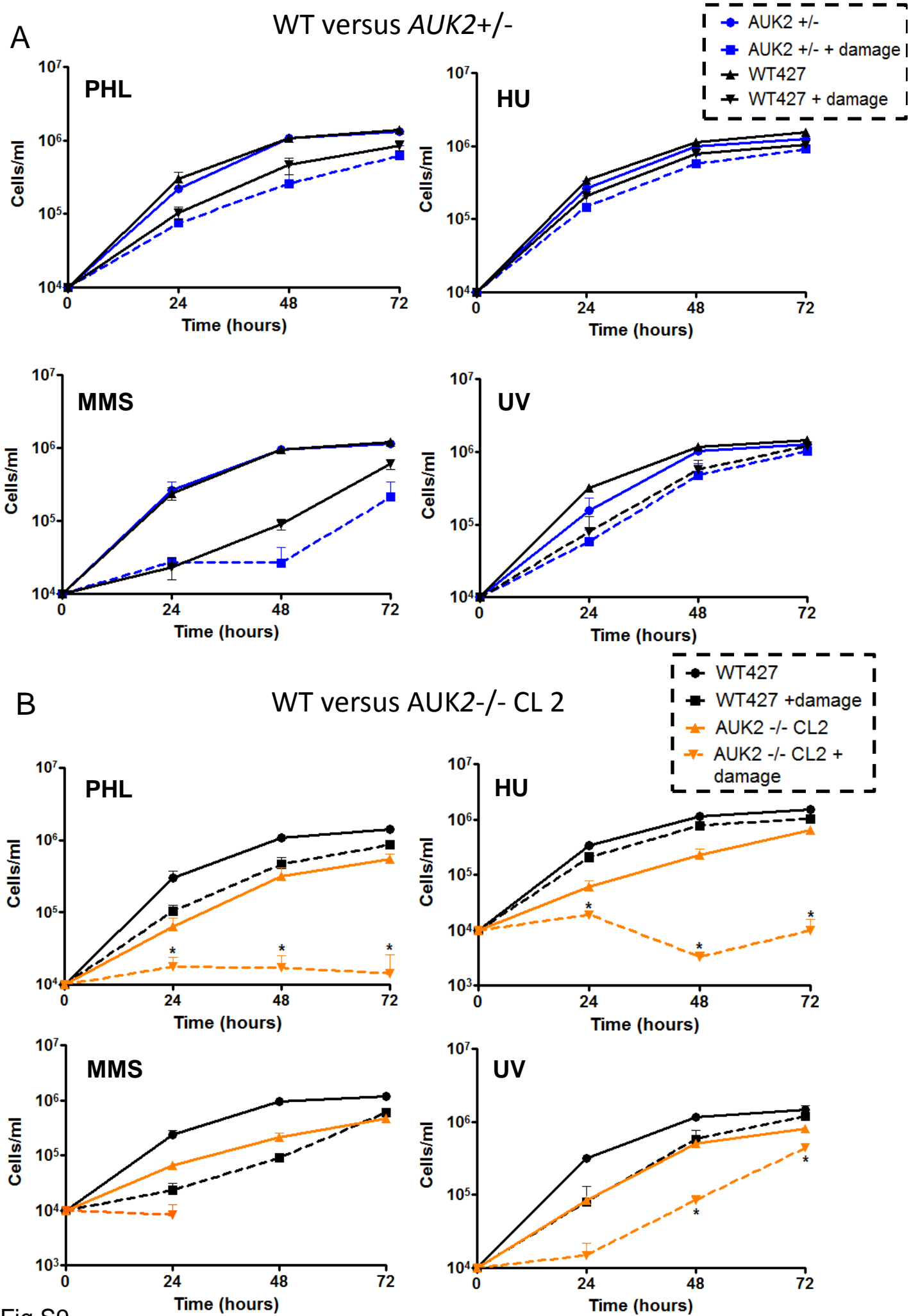

Supplement: S9 Fig — Growth curves of either (A) AUK2 heterozygous mutants (+/-) or (B) auk2 -/- null mutant clone 2 (CL2) are shown compared with wildtype (WT427) cells. In all graphs cell density was monitored every 24 hrs for 72 hrs in the presence (+) and absence of MMS (0.0003%), phleomycin (PHL; 0.1 μg.ml-1), hydroxyurea (HU; 0.6 mM; C) or after exposure to UV (1500 J/m2). All graphs show mean density from three experiments; error bars denote SEM. Significant differences are shown by * (P<0.05; Mann Whitney U test). For the -/- CL2 mutants, no cells could be detected after 24 hrs growth in MMS. (PDF) [file ppat.1006477.s015.pdf]

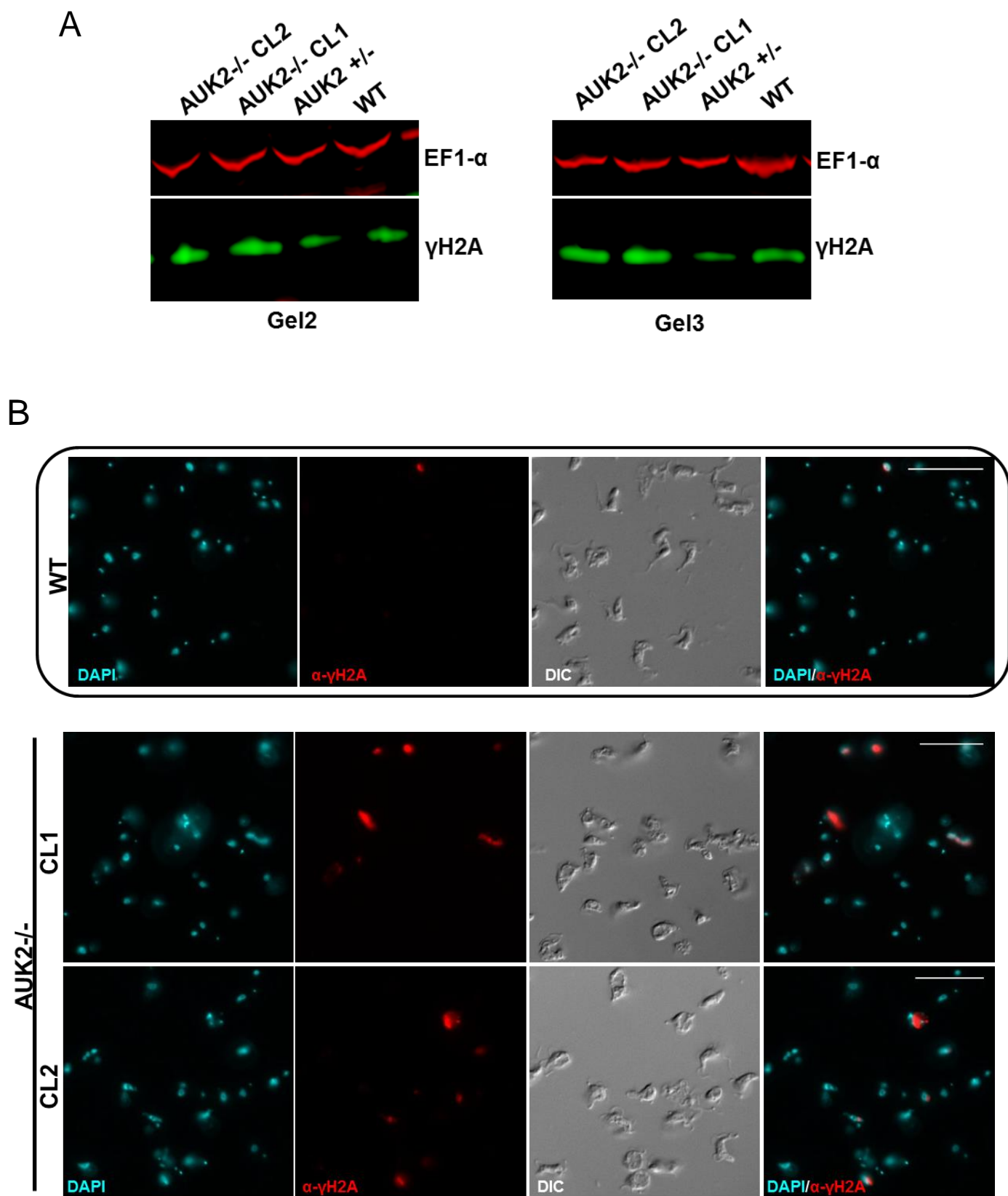

Fig.S10

Supplement: S10 Fig — A. To gels, representing independent experiments, are shown of western blot analysis of γH2A in two auk2 -/- mutants clones (CL1 and CL2) relative to an AUK+/- heterozygous mutant and wild type cells (WT427). Whole cell lysates were probed with anti-γH2A (below) and anti-EF1α (above; loading control) antisera. B. Immunofluorescence (IF) of γH2A. Cells were harvested, fixed and RAD51 localised with anti- γH2A antiserum. Representative IF images of auk2-/- mutants and WT cells are shown in which DAPI stained DNA is in blue and γH2A in red (cell morphology is shown by differential contrast imaging); the scale bar = 10 μm. (PDF) [file ppat.1006477.s016.pdf]

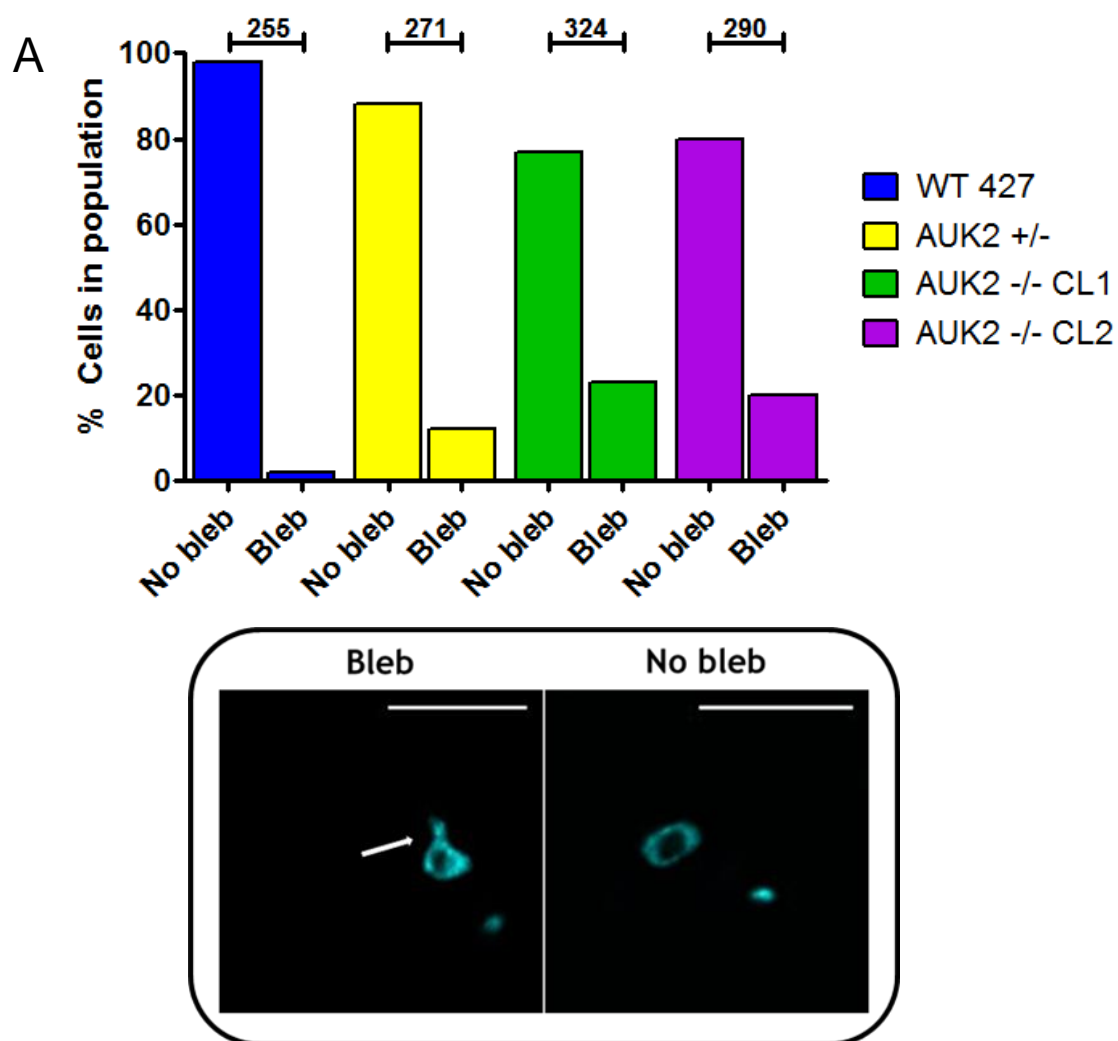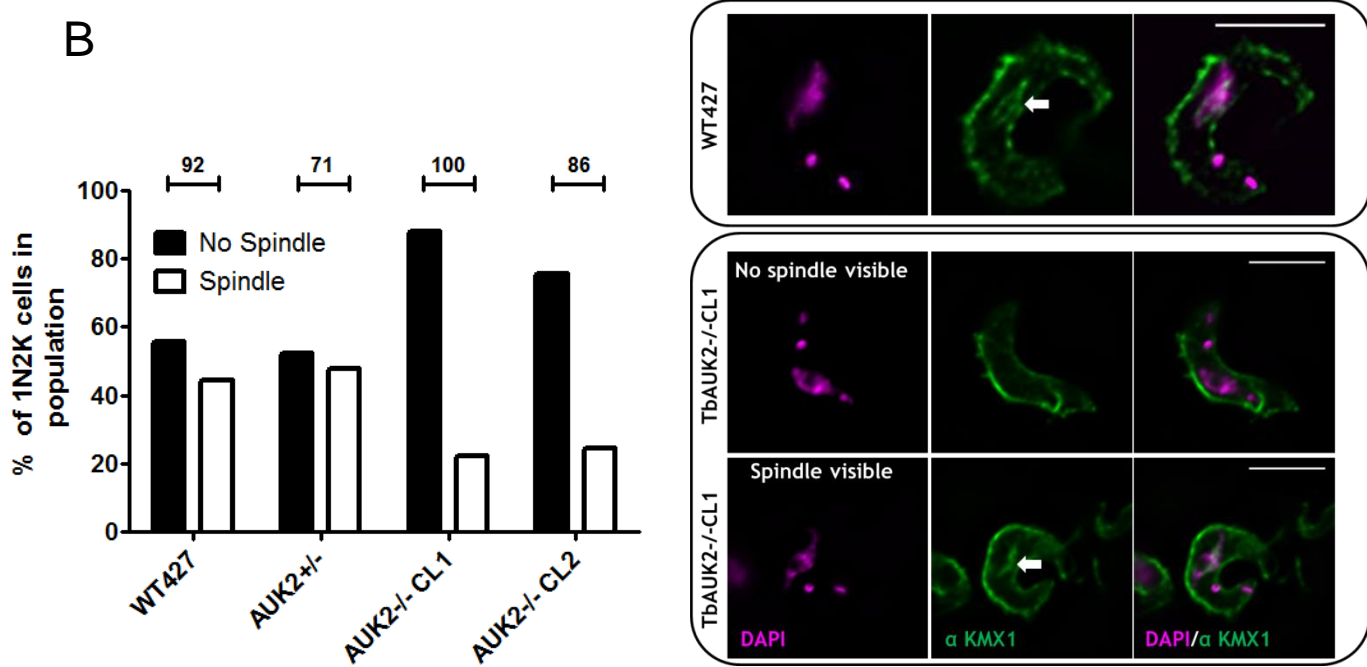

Fig.S11

Supplement: S11 Fig — A. >250 cells were imaged after DAPI staining (cyan) and examined for the presence of nuclear ‘blebs’, scoring wild type, AUK2+/-, or auk2-/- (two clones, CL1 and CL2) cells either as having a discernible ‘bleb’ or as having ‘no bleb’. Each category is expressed as a percentage of the total cell count. Below are representative examples of the two categories; scale bar = 5 μm, and the arrow indicates a bleb. B. Analysis of the mitotic spindle of 1N2K cells in WT, AUK2+/- and auk2-/- cells. Images of 1N2K cells categorised depending upon the presence (spindle) or absence of (no spindle) of a mitotic spindle, which was visualised by staining with anti-KMX-1 antiserum (green) and co-localisation with DAPI (magenta). Counts are represented as a percentage of the total cells examined; the number of cells counted is indicated above the corresponding bars. Representative images of a WT cell with an intact mitotic spindle (indicated by the white arrow), and an auk2-/- cell without a detectable spindle are shown; scale bar = 5 μm. (PDF) [file ppat.1006477.s017.pdf]

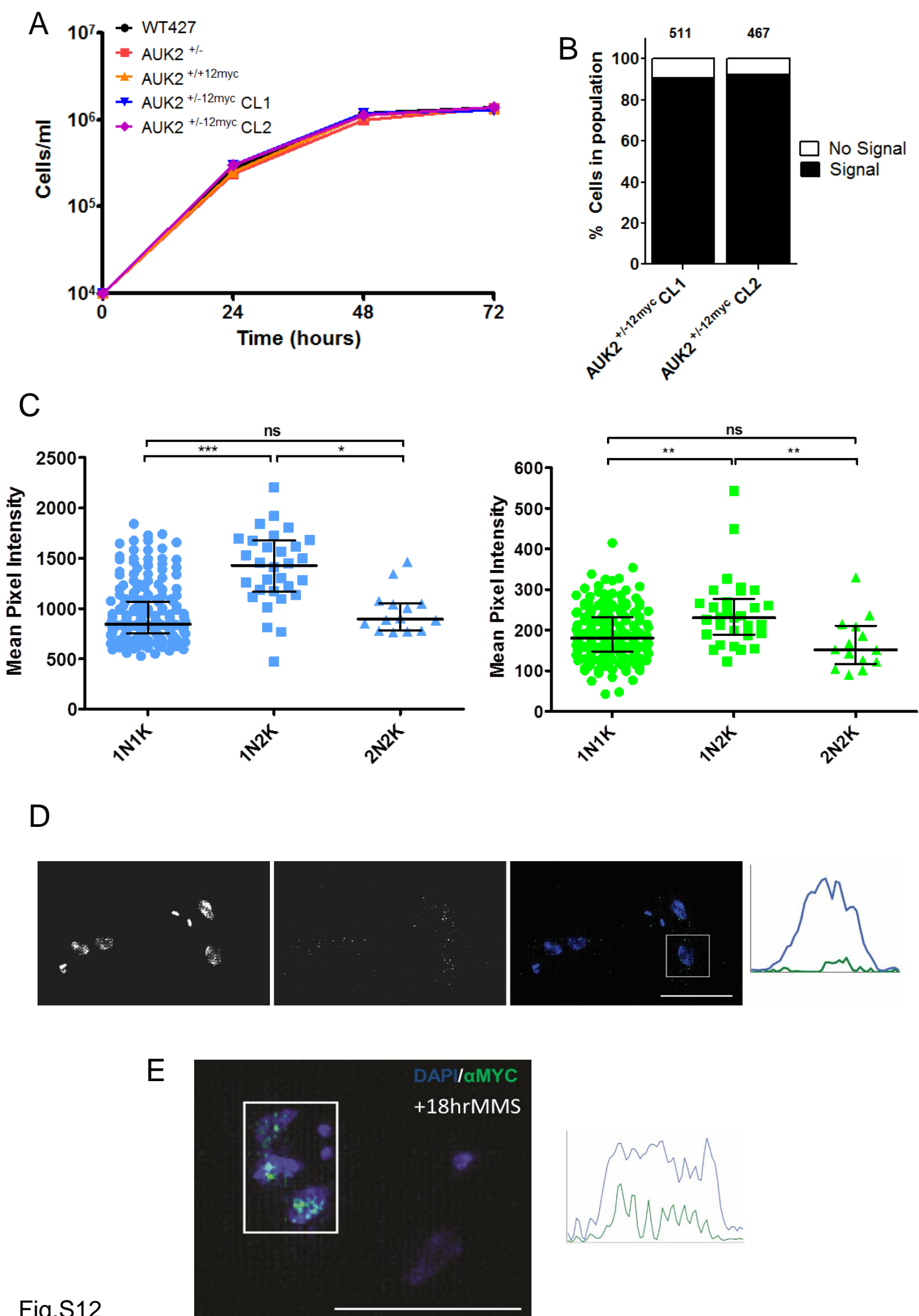

Fig.S12

Supplement: S12 Fig — A. in vitro growth analysis of AUK2+/-12myc cell lines, in which one allele of AUK2 is C-terminally fused to 12 copies of the myc epitope and the other allele is disrupted. Growth was evaluated by measuring cell density over 96 hrs and compared with wild type (WT), AUK2+/- and AUK2+/+12myc (one AUK2 allele myc-tagged, the other intact) cells. B. Quantification, after immunolocalisation of AUK2-12myc with anti-myc antiserum, of the number of AUK2+/-12myc (two clones, CL1 and CL2) cells with or without a discernible nuclear signal; the total number of cells counted is shown, and the two categories are represented as a percentage of the total. C. Intensity of nuclear DNA DAPI signal (left, blue) or anti-myc signal in immunoflouresence (right, green), was measured using ImageJ and is shown for cells with 1N1k, 1N2K and 2N2K nuclear (n) and kinetoplast (k) configurations. To perform this analysis, a region of interest (21x21 pixels) was drawn around each nucleus and the mean pixel intensity recorded (represented by a ‘dot’ on the graph). The error bars represent the median value and the interquartile range. Significance was assessed using the Kruskal-Wallis non parametric test. (*) p<0.05, (**) p<0.005 and (***) p<0.0005. n = 253 cells, n = 1. D. Super resolution images of AUK2-12myc localisation with anti-myc antiserum in 2N2K AUK2+/-12myc cells, demonstrating the lack of detectable nuclear signal. The graphs show fluorescence intensity (arbitrary units; AU) plotted for both the DAPI (blue) and anti-myc (green) signal. The white box represents the area from which the fluorescence intensity was measured (using ImageJ) to generate the graph. Fluorescence intensity (as AU) is plotted (X-axis) over distance (μm; Y-axis). Scale bar = 5 μm. E. A representative super resolution image of AUK2-12myc localisation with anti-myc antiserum after growth of AUK2+/-12myc cells for 18 hrs in 0.0003% MMS. (PDF) [file ppat.1006477.s018.pdf]
